# The genetic architecture of water-soluble protein content and its genetic relationship to total protein content in soybean

**DOI:** 10.1038/s41598-017-04685-7

**Published:** 2017-07-11

**Authors:** Dan Zhang, Haiyan Lü, Shanshan Chu, Huairen Zhang, Hengyou Zhang, Yuming Yang, Hongyan Li, Deyue Yu

**Affiliations:** 1 Collaborative Innovation Center of Henan Grain Crops, College of Agronomy, Henan Agricultural University, Zhengzhou, 450002 China; 2 The Institute of Genetics and Developmental Biology (IGDB) of the Chinese Academy of Sciences, Beijing, 100101 China; 3 Department of Biological Sciences, University of North Carolina at Charlotte, Charlotte, NC 28223 USA; 4 National Center for Soybean Improvement, National Key Laboratory of Crop Genetics and Germplasm Enhancement, Nanjing Agricultural University, Nanjing, 210095 China

## Abstract

Water-soluble protein content (WSPC) is a critical factor in both soybean protein quality and functionality. However, the underlying genetic determinants are unclear. Here, we used 219 soybean accessions and 152 recombinant inbred lines genotyped with high-density markers and phenotyped in multi-environments to dissect the genetic architectures of WSPC and protein content (PC) using single- and multi-locus genome-wide association studies. In the result, a total of 32 significant loci, including 10 novel loci, significantly associated with WSPC and PC across multi-environments were identified, which were subsequently validated by linkage mapping. Among these loci, only four exhibited pleiotropic effects for PC and WSPC, explaining the low correlation coefficient between the two traits. The largest-effect WSPC-specific loci, *GqWSPC8*, was stably identified across all six environments and tagged to a linkage disequilibrium block comprising two promising candidate genes *AAP8* and *2 S albumin*, which might contribute to the high level of WSPC in some soybean varieties. In addition, two genes, *Glyma*.*13G123500* and *Glyma*.*13G194400* with relatively high expression levels at seed development stage compared with other tissues were regarded as promising candidates associated with the PC and WSPC, respectively. Our results provide new insights into the genetic basis of WSPC affecting soybean protein quality and yield.

## Introduction

Soybean is an important legume because of high protein with a nutritionally balanced amino acid profile in seeds, therefore soybean seeds are commonly used as a prime source of vegetable protein worldwide^1^. Soybean protein usually is fairly soluble in water, and only water-soluble protein can be processed and utilized in traditional soyfoods^2^. Therefore, water-soluble protein content (WSPC) is a critical factor in both food quality and the production of isolated soybean proteins. At present, soybean proteins have been widely used in many protein-based food formulations mainly because of their functional properties, i.e., solubility, which is a critical factor in the acceptability of beverages, additives, and fortifier. Thus far, many approaches have been carried out to improve the solubility of soybean, such as physical modification^3, 4^, chemical modification^5^, and enzymatic modification^6^. Although the processing of soybean protein in these manners is effective, it increases the cost of the finished product and is not a sustainable approach.

Soybean WSPC and total protein content (PC) are complex quantitative traits controlled by multiple genes, some might have small effects^7^. Our previous study has shown a substantial natural variation in soybean WSPC, ranging from 10 to 45% in a panel containing diverse soybean accessions^8^. Moreover, in the breeding practice, we found that some high-PC soybean varieties contain a low level of WSPC, whereas, some varieties contain a moderate level of PC but relatively high WSPC. In addition, high-resolution DNA markers are helpful in fine mapping of quantitative trait loci (QTLs) controlling the complex traits, such as WSPC. The subsequent genotype selection assisted by marker-assisted selection (MAS) would facilitate the development of soybean cultivars with improved WSPC. However, the way how soybean WSPC is genetically controlled remains largely unknown. Our current knowledge of WSPC is mainly based on the genetic studies of its correlated trait, PC. Thus, dissecting the genetic architecture of soybean WSPC and identifying the genetic relationship between WSPC and PC are urgently needed.

In the past decades, tremendous efforts have been made to dissect the genetic basis of soybean protein related traits. More than 100 QTLs related to soybean PC have been reported, including its components (7S and 11S) (http://www.soybase.org/), but most of these QTLs/genes were intensively focused on soybean total protein. Thus far, only several genes related to soybean PC have been cloned and functionally identified, but no WSPC-related genes have been reported. For example, seven major glycinin genes (*G1* to *G7*) encode glycinin subunits, with Group-1 (*G1*, *G2*, *G3*) showing higher expression level^9, 10^. In addition, some studies showed that the amino acid permease in *Vicia narbonensis* and pea can increase seed storage proteins^11, 12^. Recently, QTLs underlying WSPC have been identified in soybean^8, 13^. Despite a preliminary understanding of the soybean WSPC obtained, the molecular basis of natural variation in WSPC biosynthesis has not been fully elucidated because the QTL resolution is limited by the low density of molecular markers used in these studies. Additionally, the genetic relationship between WSPC and PC is unclear, resulting in difficulty in the improvement of the soybean cultivars with increased content of both WSPC and PC by MAS. Thus, a comprehensive genetic study is needed to determine the extent of genetic relevance between soybean WSPC and PC.

Genome-wide association studies (GWAS) using high-density DNA markers offer an opportunity to dissect the genetic architecture of complex traits in soybean. Compared with the QTL linkage mapping approach, GWAS can greatly increase the range of detection of natural variation, the number of genome-wide significant loci, and even QTL resolution for complex agronomic traits. By applying GWAS, many important QTLs could be narrowed down and associated candidate genes could be identified^14, 15^. Recently, a soybean collection containing 367 diverse accessions has been genotyped using a high-throughput NJAU 355 K SoySNP array, which provides a high-resolution of genome-wide markers facilitating GWAS of complex traits in soybean^16^.

In this study, we conducted a high-resolution GWAS of soybean WSPC and PC within 219 diverse association accessions (a large portion of the 367 diverse accessions) genotyped with NJAU 355 K SoySNP array to dissect the underlying genetic architecture of WSPC and PC in soybean. In addition, to fully understand the genetic architecture of WSPC and its genetic relationship to PC at the QTL level, epistatic GWAS (EGWAS) were also presented in this study as previously described^17, 18^. Moreover, a recombinant inbred lines (RILs) population whose parents were selected from the association panel was used to validate the significant signals identified in GWAS. Candidate genes within these significant association loci that were potentially involved in the regulation of WSPC and PC were also predicted.

Our results identified 32 loci distributed over different chromosomes significantly associated with WSPC and PC in at least three or more environments, and only four regions exhibited pleiotropic effects for WSPC and PC. This observation may explain low correlation coefficient between the two traits as observed in phenotypic correlation. Moreover, QTLs associated with soybean WSPC and PC exhibit a moderate level of genetic sharing, suggesting these two traits may be under differential directional selection during soybean domestication and improvement. Those WSPC-specific loci might be responsible for high WSPC in the low-protein soybean varieties. G*qWSPC8* is a highly significant major-effect locus that specifically affects WSPC. This region contained two candidate genes encoding seed storage 2S albumin proteins (*Glyma*.*08G112300*) and amino acid permease (*AAP8*, *Glyma*.*08G113400*), which may be responsible for the high WSPC in soybean. In addition, other candidate genes, such as *Glyma*.*13G123500* and *Glyma*.*13G194400* with relatively high expression levels at seed development stage were also regarded as promising candidates associated with the PC and WSPC, respectively.

## Results and Discussion

### WSPC and PC exhibited significant phenotypic variation

**Figure 1:**
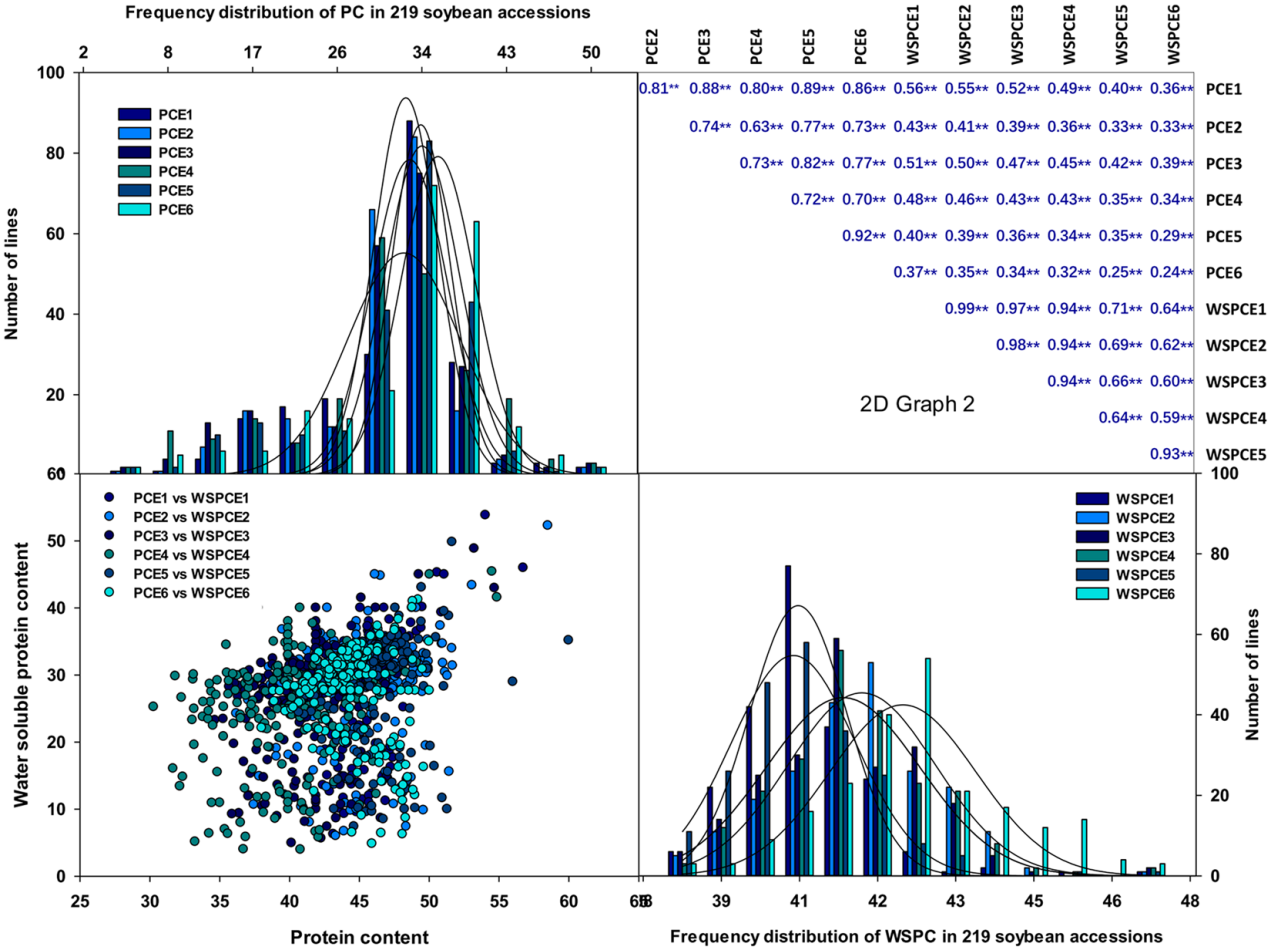
Phenotypic analysis of protein content (PC) and water-soluble protein content (WSPC) in the 219 soybean accessions. The histograms on the diagonal show the phenotypic distribution of each trait across six environments. The values above the diagonal are pairwise correlation coefficients between traits, and the plots below the diagonal are scatter plots of compared traits. PCE1-E6, denote the protein content in six different environments; WSPCE1-E6, denote the water-soluble protein content in the corresponding environments.

The selection of appropriate mapping populations genotyped with saturated markers is important for the dissection of mapped QTLs and further understanding the genetic architecture^19^. In this study, the 219 accessions were collected from three different ecological habitats, which represent all geographic ranges of soybean cultivation in China, suggesting that this panel is representative and is expected to contain a great level of genetic variation. For example, a majority of accessions (approximately 175 accessions) from this panel have been used to identify QTLs associated with yield^20^, seed shape traits^21^, phosphorus efficiency, and soybean protein^8^, suggesting that this collection might contain diverse phenotypic variation in complex quantitative traits of soybean. As expected, a great level of genetic variation in WSPC and PC were observed in its expanded panel (219 accessions) that was used in the present study (Table S1, Fig. 1 and Figure S1). Similarly, the RIL population whose parents were from the association panel also exhibited significant variation in biological yield^22^, and responses to low-P stress^23, 24^. The two parents also differed considerably for the PC and WSPC across environments^8^. On the other hand, the availability of the dense genome-wide markers for both populations would be also beneficial in enhancing mapping resolution. For example, the association panel has been genotyped with approximate 355,000 SNP markers^16^, which is approximately ten times more than the marker density (approximate 30,000 SNPs) that was used in recent GWAS studies in soybean^17, 25^. These high-coverage markers here can increase the resolution of association mapping (one SNP/3.3 kb). In addition, we have used the RIL population to construct a high-density genetic map with 6,159 SNP markers, with an average distance of 0.49 cM between adjacent markers^24^. Thus, the SNP datasets, the association panel, and the segregating RIL population are appropriate to dissect the genetic basis of WSPC, PC, and other complex traits.

Both mapping populations exhibited significant phenotypic variation in PC and WSPC across environments. As shown in Table S1, the means, standard deviation, range, skew, and the broad-sense heritability of PC and WSPC were calculated. The mean PC for the individual accessions in the GWAS population ranged from 30.3 to 57.1%, and the maximum value for WSPC can reach 46.8% (E3), which was approximately eight times higher than the minimum value (6.3%, E1) (Table S1). In RIL population, the phenotypic values ranged from 37.4–46.5% for PC and 22.7–38.8% for WSPC. The distribution of both traits for these GWAS accessions is roughly normal, and the range exceeds those that were observed among the RILs at both sides of the distribution (Table S1 and Fig. 1). More extensive natural variation may be an advantage for GWAS, which can break the limitation of allelic diversity in a segregating population^26^. Thus, both PC and WSPC exhibited wide phenotypic variation among this association panel across different environments and displayed very high genetic diversity (Table S1, Figs 1 and S1).

The high correlation coefficient of phenotypic value for a trait between different years (Figs 1 and S1) indicates the high quality of the phenotypic dataset. However, the correlation coefficient between the PC and WSPC is relatively low in the same year, although they are significant at the 0.01 level in all of the environments (Figs 1 and S1). In addition, the correlation between PC and WSPC: PC was not significant (*r* = 0.12, *P* = 0.082).

### Significant loci associated with PC and WSPC

**Figure 2:**
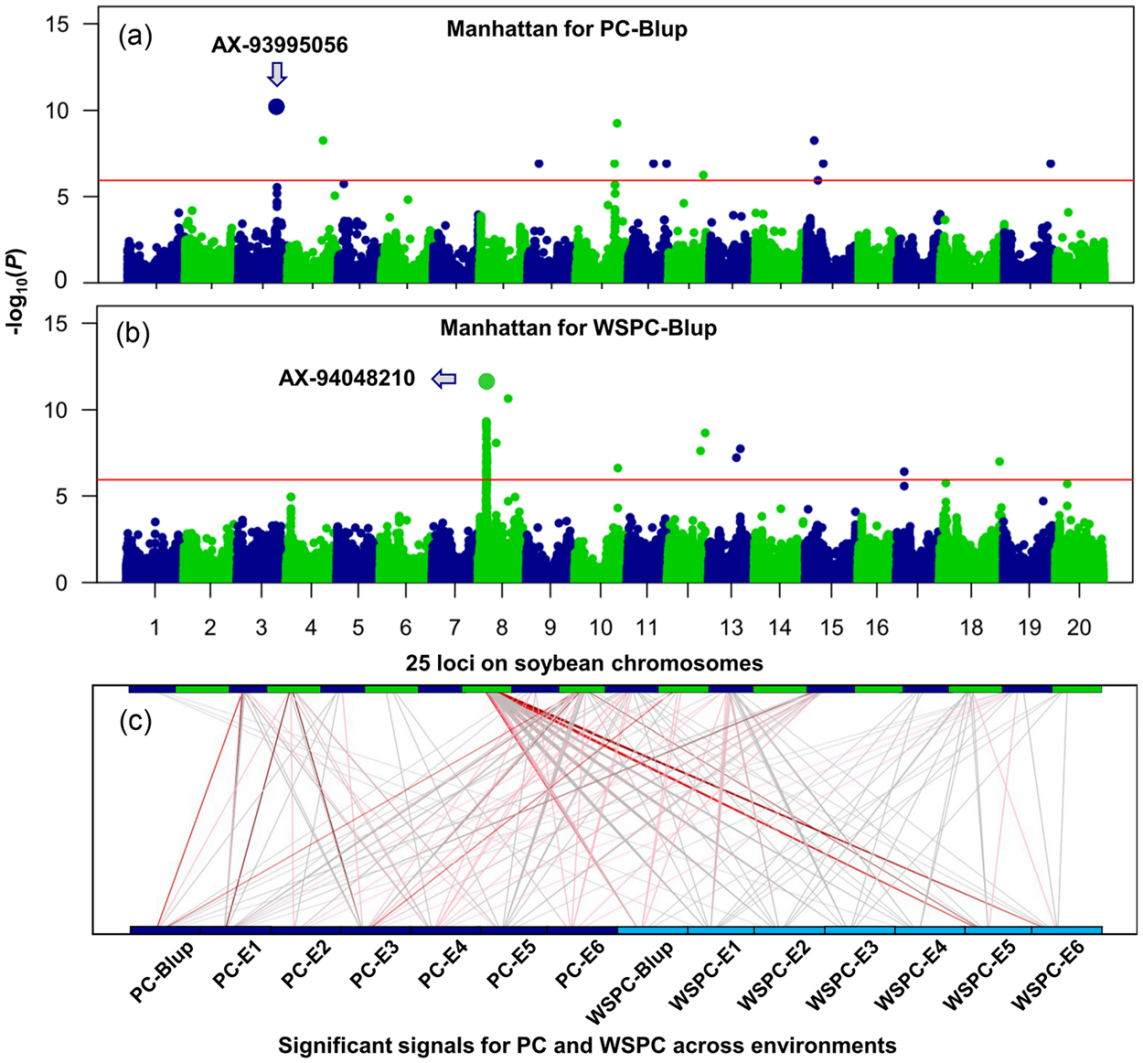
Genetic architecture of soybean protein content (PC) and water-soluble protein content (WSPC). (**a**) and (**b**) Manhattan plot for the BLUP of soybean PC and WSPC across six environments by genome-wide association mapping. Red horizontal lines depict the Bonferroni-adjusted significance threshold (*P* < 4.95 × 10^−6^). The x axis shows the 20 soybean chromosomes, and the y axis shows the significance expressed as −log_10_ *P* value. (**c**) Associations between 25 loci aligned on the upper boundary and 14 phenotype values (contain two traits across six environments and their BLUP) aligned on the lower boundary. Positions of loci correspond to the above panel. Deep red, red, pink, and gray lines represent significant associations between SNPs and phenotype value with threshold levels of *P* < 1.0 × 10^−11^, *P* < 1.0 × 10^−9^, *P* < 1.0 × 10^−7^, *P* < 1.0 × 10^−5^, respectively.

Of 292,053 high-quality SNPs, firstly, we selected a total of 201,994 polymorphic SNPs with minor allele frequency (MAF) ≥0.05 were used for GWAS using GAPIT package. For one trait, lead SNPs less than or around 2-Mb were considered as caused by one common gene^27^. Base on this rule, our GWAS identified a total of 25 loci (15 for PC and 10 for WSPC) comprising 589 SNP signals significantly associated with WSPC and PC at least three or more environments (containing their BLUP) at *P* < 4.95 × 10^−6^ (Table 1 and Fig. 2a,b and c). The full list of significant SNPs associated with both traits across six environments and the BLUP is presented in Tables S2 and S3. As shown in the quantile-quantile and Manhattan plots for BLUP of PC and WSPC (Fig. 2a,b) and individual environment (Figures S2 and S3), we identified positive associations significant for PC and WSPC after using mixed linear model in accounting for population structure and familial relatedness^28^. Of 25 loci, more than half (18) of the loci identified by GWAS co-localized with the previously-identified QTLs associated with seed protein or protein components (Table 1). Moreover, the genomic regions of almost all these previously-identified QTLs were significantly narrowed (Table 1). In addition, we also identified seven loci (*GqPC4*-*1*, *GqPC5*, *GqPC6*, *GqPC11*-*2*, *GqPC15*-*2*, *GqWSPC11*-*2*, *GqWSPC18*-*2*) that were not found in previous reports, representing novel loci underlying soybean protein content. These results indicated that GWAS is a powerful strategy in the genome-wide identification of phenotype-genotype associations^23, 29^. Moreover, the highly diverse association panel and saturated genome-wide DNA markers are both critical factors improving the mapping resolution. Both advantages enable GWAS-based mapping to finely map previously-identified QTLs and detect novel loci.

**Table 1:** Loci significantly associated with soybean protein content (PC) and water soluble protein content (WSPC) and the candidate genes.

QTL	Chr^**a**^	Rep. SNP^b^	Pos. (bp)^c^	No. sig.^d^	*R* ^*2*^	Traits-Environments^e^	Related QTL^f^	Candidate genes^g^	Annotations^h^
*GqPC3*	3	AX-93995056	37,773,722	18	0.229	E1, E2, E3, E4, E5, BLUP	Seed protein 21-9, Seed Leu 1-7	*Glyma*.*03G156100*	Glycinin A2B1a precursor
*GqPC4*-*1*	4	AX-94274580	34,743,951	7	0.292	E1, E2, E3, E4, E5, E6, BLUP	/	*/*	Family not named
*GqPC4*-*2*	4	AX-93920058	46,200,673	4	0.227	E3, E4, E5, E6	Seed protein 3-3, Seed protein 4-1,	*Glyma*.*04G157500*	Leucine-rich repeat protein kinasefamily protein
*GqPC5*	5	AX-94012201	5,637,601	4	0.174	E1, E2, E3, BLUP	/	*Glyma*.*05G070600*	Nitrate transporter
*GqPC6*	6	AX-93731783	26,098,086	3	0.269	E4, E5, E6	/	*Glyma*.*06G220300*	Phospholipid acyltransferase
*GqPC9*	9	AX-93764734	11,138,977	6	0.258	E2, E3, E4, E5, E6, BLUP	Seed acidic fraction 1-2, Seed Thr 1-4	*Glyma*.*09G087200*	inositol transporter
*GqPC10*	10	AX-93933852	40,504,375	33	0.254	E1, E2, E3, E4, E5, E6 BLUP	Seed protein 27-5	*Glyma*.*10G177000*	RmlC-like cupins superfamily protein
*GqPC11*-*1*	11	AX-94089116	24,782,059	6	0.258	E3, E4, E5, E6, BLUP	Seed protein 25-1, 25-2	*/*	Family not named
*GqPC11*-*2*	11	AX-93795201	37,461,551	12	0.259	E1, E3, E4, E5, E6, BLUP	/	*Glyma*.*11G234600*	Transmembrane amino acid transporter
*GqPC12*	12	AX-93804391	34,037,114	5	0.260	E1, E2, E3, E5, BLUP	Seed protein 5-2, 33-1, 21-10	*Glyma*.*12G179700*	Serine carboxypeptidase S28 family protein
*GqPC13*	13	AX-94109235	23,091,289	3	0.220	E1, E3, E4	Seed protein 3-7	*Glyma*.*13G123500*	Glycinin A3B4 subunits
*GqPC14*	14	AX-93822697	7,715,347	3	0.032	E1, E2, E6	Seed Protein 1-2	*Glyma*.*14G086300*	Asparaginase
*GqPC15*-*1*	15	AX-93649790	7,681,400	7	0.292	E1, E3, E4, E5, E6, BLUP	Seed protein 5-1, 3-6, 4-6	*Glyma*.*15G098100*	Beta-amylase
*GqPC15*-*2*	15	AX-93837099	11,235,816	6	0.226	E1, E3, E4, E5, E6, BLUP	/	*Glyma*.*15G138200*	Rab5-interacting family protein
*GqPC15*-*3*	15	AX-94136114	16,417,543	6	0.258	E1, E3, E4, E5, E6, BLUP	Seed Lys 1-2, Seed Tyr 1-3	*Glyma*.*15G175200*	Zinc finger WD40 repeat protein
*GqPC19*	19	AX-94196006	47,615,818	10	0.267	E1, E3, E4, E5, E6, BLUP	Seed protein 2-2,16-2	*Glyma*.*19G236600*	7S globulin precursor
*GqWSPC1*	1	AX-93961814	9,078,593	3	0.095	E2, E4	Seed protein 3-5	*/*	Family not named
*GqWSPC7*	7	AX-94283226	3,484,506	4	0.049	E1, E2, E3, BLUP	Seed protein 36-33	*Glyma*.*07G041800*	RNA polymerase III subunit RPC6
*GqWSPC8*	8	AX-94048210	8,643, 359	469	0.193	E1, E2, E3, E4, E5, E6, BLUP	Seed protein 26-1, 30-4	*Glyma*.*08G112300*	Seed storage 2S albumin protein
								*Glyma*.*08G113400*	Amino acid permease
*GqWSPC10*	10	AX-93933852	40,504,375	33	0.130	E1, E2, E4, E5, E6, BLUP	Seed protein 27-5	*Glyma*.*10G177000*	RmlC-like cupins superfamily protein
*GqWSPC11*-*2*	11	AX-93795201	37,461,551	19	0.089	E1, E2, E3, E4, E5, E6	/	*Glyma*.*11G234600*	Transmembrane amino acid transporter
*GqWSPC12* - *1*	12	AX-94268787	2,583,421	4	0.038	E1, E2, E3, BLUP	/	*Glyma*.*12G034900*	Auxin responsive protein
*GqWSPC12*-*2*	12	AX-93805526	36,952,663	5	0.123	E1, E2, BLUP	Seed protein 5-2, 33-1, 21-10	*Glyma*.*12G205100*	Concanavalin A-like/Legume lectin domain
*GqWSPC13*	13	AX-94112235	30,988,071	19	0.110	E1, E2, E3, E4, E5, E6, BLUP	Seed protein21-6, 33-2	*Glyma*.*13G194400*	Albumin 1 gene
*GqWSPC15*	15	AX-93649393	48,823,741	3	0.058	E1, E2, E3	/	*Glyma*.*15G258100*	Lob domain-containing protein 4
*GqWSPC16*	16	AX-94150758	28,496,401	6	0.030	E2, E3, E4, BLUP	/	*Glyma*.*16G130900*	Proline dipeptidase
*GqWSPC17*	17	AX-94156641	7,962,112	10	0.123	E1, E2, E3, BLUP	Seed Leu 1-3	*Glyma*.*17G097800*	Aldehyde dehydrogenase
*GqWSPC18*-*1*	18	AX-93870261	6,745,767	6	0.080	E1, E2, E3, E4, E5, E6, BLUP	Seed protein 26-8, 26-14	*Glyma*.*18G071900*	Amino acid permease
*GqWSPC18*-*2*	18	AX-94170844	17,076,547	4	0.083	E2, E4, E5	/	*Glyma*.*18G126900*	GDSL-like Lipase
*GqWSPC18* - *3*	18	AX-94182257	56,190,147	5	0.011	E1, E3, BLUP	Seed protein 30-10	*Glyma*.*18G276800*	Amino acid transporter 6
*GqWSPC19*	19	AX-94196006	47,615,818	12	0.079	E1, E2, E5, BLUP	Seed protein 2-2,16-2	*Glyma*.*19G236600*	7S globulin precursor
*GqWSPC20*	20	AX-94198333	4,049,700	5	0.022	E2, E3, E4	Seed protein 11-1	*Glyma*.*20G031900*	DNA replication licensing factor

^a^Chromosome; ^b^the representative SNP with the minimum P value; ^c^representative SNP position on soybean genome assembly *Glycine max* Wm82.a1.v1.1 (www.phytozome.net); ^d^the number of significant association signals detected in the region; ^e^the significant signals were associated with the traits across different environments; ^f^Previously reported protein-related QTL in SoyBase (http://www.soybase.org/); ^g,h^Genes ID and annotated in *Glycine max* Wm82.a2.v1 (www.phytozome.net), and NCBI RefSeq gene models in SoyBase (www.soybase.org) were used as the source of candidate genes. The QTNs with bold type were identified simultaneously by single- and multi-locus GWAS methods, and the underlined QTNs were detected only by multi-locus GWAS methods.

**Figure 3:**
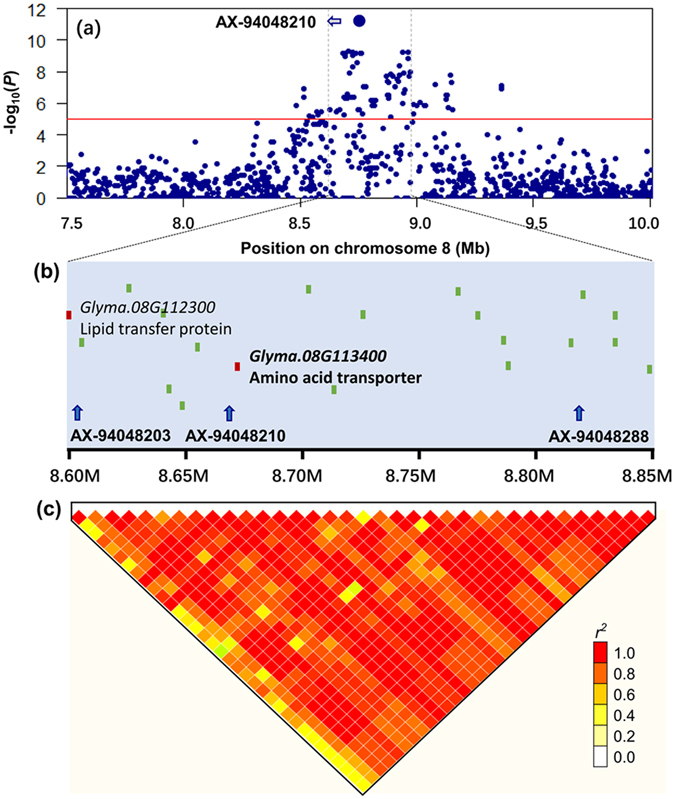
Associations, genomic locations and the pattern of pairwise LD of SNPs associated with water-soluble protein content (WSPC) on chromosome 8. (**a**) A 2.5-Mb region of the major-effect quantitative trait loci (*GqWSPC8*) harboring the peak SNP, AX-94048210 on chromosome 8. The most significantly associated SNP is shown with a big blue dot. Red horizontal lines depict the Bonferroni-adjusted significance threshold (*P* < 4.95 × 10^−6^). The x axis shows the genomic position, and the y axis shows the significance expressed as −log_10_ *P* value. (**b**) Soybean genome region around the SNP marker, AX-94048210 on chromosome 8, whose position is indicated by a vertical gray dashed line (0.25-Mb) on the top panel. (**c**) The extent of linkage disequilibrium (LD) in the regions based on pairwise *r* ^*2*^ values. The *r* ^*2*^ values are indicated using the color intensity index. Heatmap showing LD between each pair of markers that passed the Bonferonni threshold in GWAS.

A total of 15 loci associated with PC were identified, and the average phenotypic variation explained by each loci ranged from 17.4% (G*qPC5*) to 29.2% (G*qPC15*-*1*) (Table 1). This result suggests that soybean PC is a complex trait and controlled by many loci with relative larger effect. For WSPC, a total of 10 loci were detected, explaining 7.9–19.3% of the average phenotypic variation (Table 1). Compared with PC, most of the loci (except *GqWSPC8*) for WSPC explained <10% of the phenotypic variation (Table 1 and Table S3), suggesting that the genetic architecture of WSPC is relatively more complex than we were expected and WSPC might be controlled by a major QTL plus many relative small-effect QTLs. Notably, *GqWSPC8* on Gm08, comprising 427 significant SNP signals affecting soybean WSPC, represented the strongest associated hotspot region and was detected across all six environments. This QTL explained 17.9% of the average phenotypic variation (Figs 2 and 3a).

The above GWAS was single locus-based with multiple tests, and Bonferroni correction is adopted to reduce false discovery rate. Although stringent, this correction might miss some important loci. To maximally capture the important variation underlying PC and WSPC, we also adopted several multi-locus-based GWAS methods to analyze the above datasets, such as ISIS EM-BLASSO and mrMLM, and the results were compared with GAPIT results. In the result, the major loci (*GqWSPC8*, *GqWSPC19*) detected by GAPIT could also be identified using two multi-locus methods (Tables 1, S2 and S3). For example, the locus (*GqWSPC8*) on Gm08 for WSPC could be identified in all the environments and their BLUP. Meanwhile, seven additional loci (Table 1) were identified by the two multi-locus methods (Tables 1, S2 and S3).

### Epistatic association analyses

Previous studies associated with marker-assisted and genomic selections have shown that epistasis should be considered in soybean breeding^17, 30, 31^. In this study, we found that the average values of PC and WSPC in E1 (32.1°N 118.4°E) in southern China was less than those in northern China (34.8°N 113.6°E) (Table S1). This observation indicates that the geographic environments might be an important factor affecting soybean protein related traits^32^. Therefore, additive QTL effects might only explain a limited proportion of the heritability for complex traits^33^. The interaction between genetic variants may be an important source of the missing heritability. To understand whether epistatic associations occurred between PC and WSPC loci across all environments, we performed epistatic GWAS (EGWAS).

**Figure 4:**
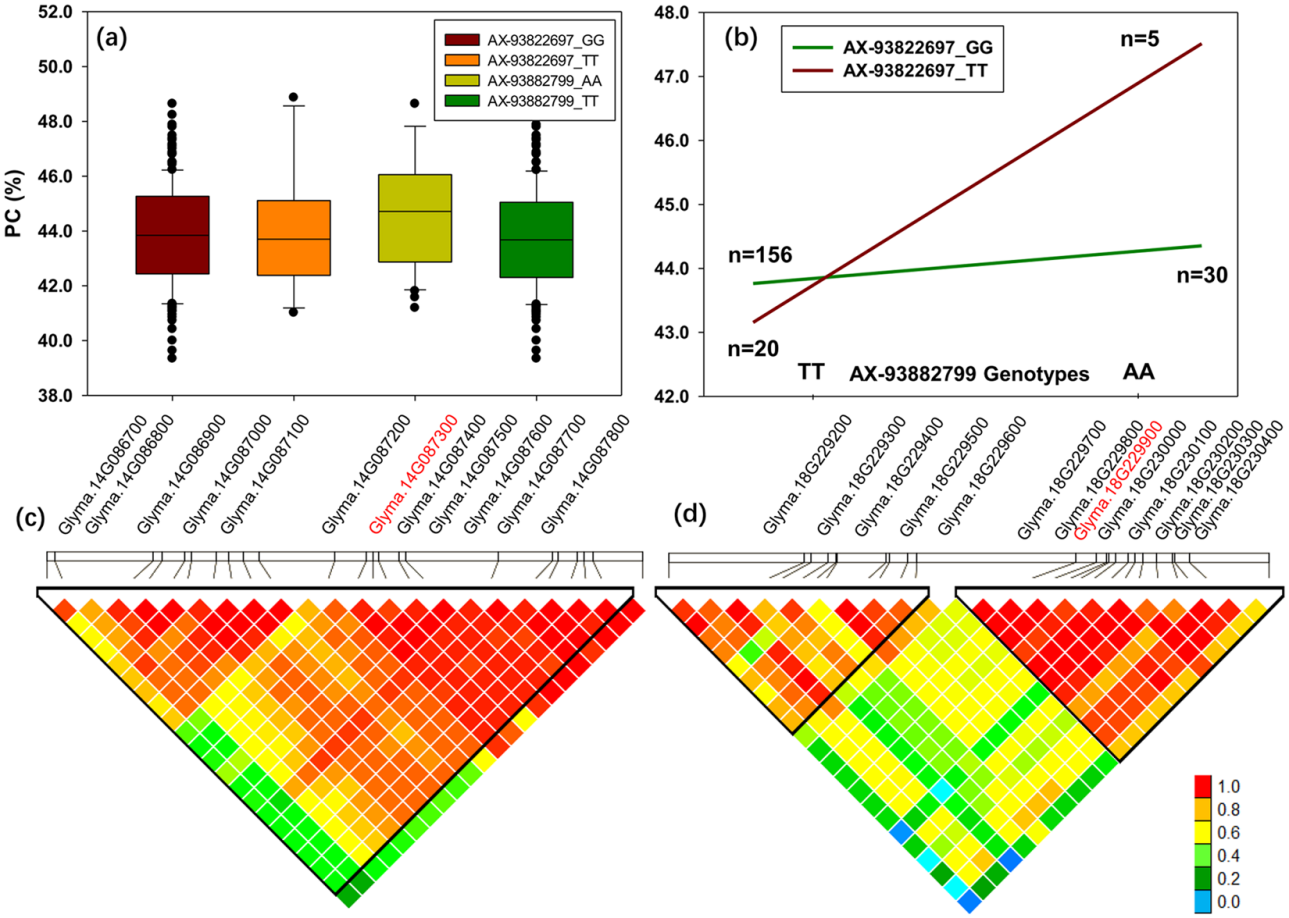
Epistatic interaction between AX-93822697_T_A and AX-93952504 _G_T associated with PC, and candidate gene for each SNP locus. (**a**) Box plot of PC based on different genotypes in soybean accessions. (**b**) Phenotypic differences between genotype combinations of the two SNP. (**c**) and (**d**) Candidate genes for AX-93822697_T_A and AX-93952504 _G_T loci, respectively. The proposed causal genes are indicated in red. The bottom panel depicts the extent of linkage disequilibrium in the regions based on pairwise *r* ^*2*^ values. The *r* ^*2*^ values are indicated using the color intensity index shown.

Because high marker density may result in prohibitive computing time, only the additive SNPs (*P* < 1.0 × 10^−4^) for each trait at each environment were selected as representatives and tested for epistatic interactions. In the result, 14 and 36 epistatic interactions were found to be associated with PC and WSPC, respectively, in at least two environments (Table S4). We found that most of the interactions detected occurred between the SNPs on different chromosomes. For example, the interaction between AX-93822697_T_A (MAF = 0.16) on Gm14 and AX-93952504 _G_T (MAF = 0.13) on Gm18 were associated with PC in three environments, having the strongest epistatic effect (*P* = 1.77 × 10^−29^) (Table S4 and Fig. 4). Individually, each SNP was not significantly associated with PC (Fig. 4a). However, accessions with the genotype TA combination (47.5%, T*A) of the epistatic loci has the highest PC of 10%, more than that with the TT combination (43.1%) (T*T) (Fig. 4b). A further examination of all four genotype combinations for the epistatic loci revealed that they were significantly different from each other, implying selection based on the epistatic effect is still effective. However, selection using AX-93822697_T_A or AX-93952504 _G_T alone may have no effect on soybean PC if both are segregating in a population.

For WSPC, it is important to note that 19 interactions shared a common locus at the 8.6-Mb genomic region on Gm08 containing six significant SNPs (AX-94048155, AX-93751882, AX-94048176, AX-93751901, AX-93751903 and AX-94048210) within a high LD block (*r* ^2^ = 0.62–0.94). This LD block was repeatedly detected for WSPC in different environments by GWAS and EGWAS (Tables 1 and S4), implying that this genomic region has both additive and epistatic effects to WSPC. The above results suggest that a complex network of genetic effects involved in the regulation of PC and WSPC in soybean. Similar complex effects affecting soybean protein and oil were also previously observed in soybean^34^ and other species such as maize^35^, rice^36^, and pea^37^.

### GWAS signals were supported by QTL linkage mapping

**Figure 5:**
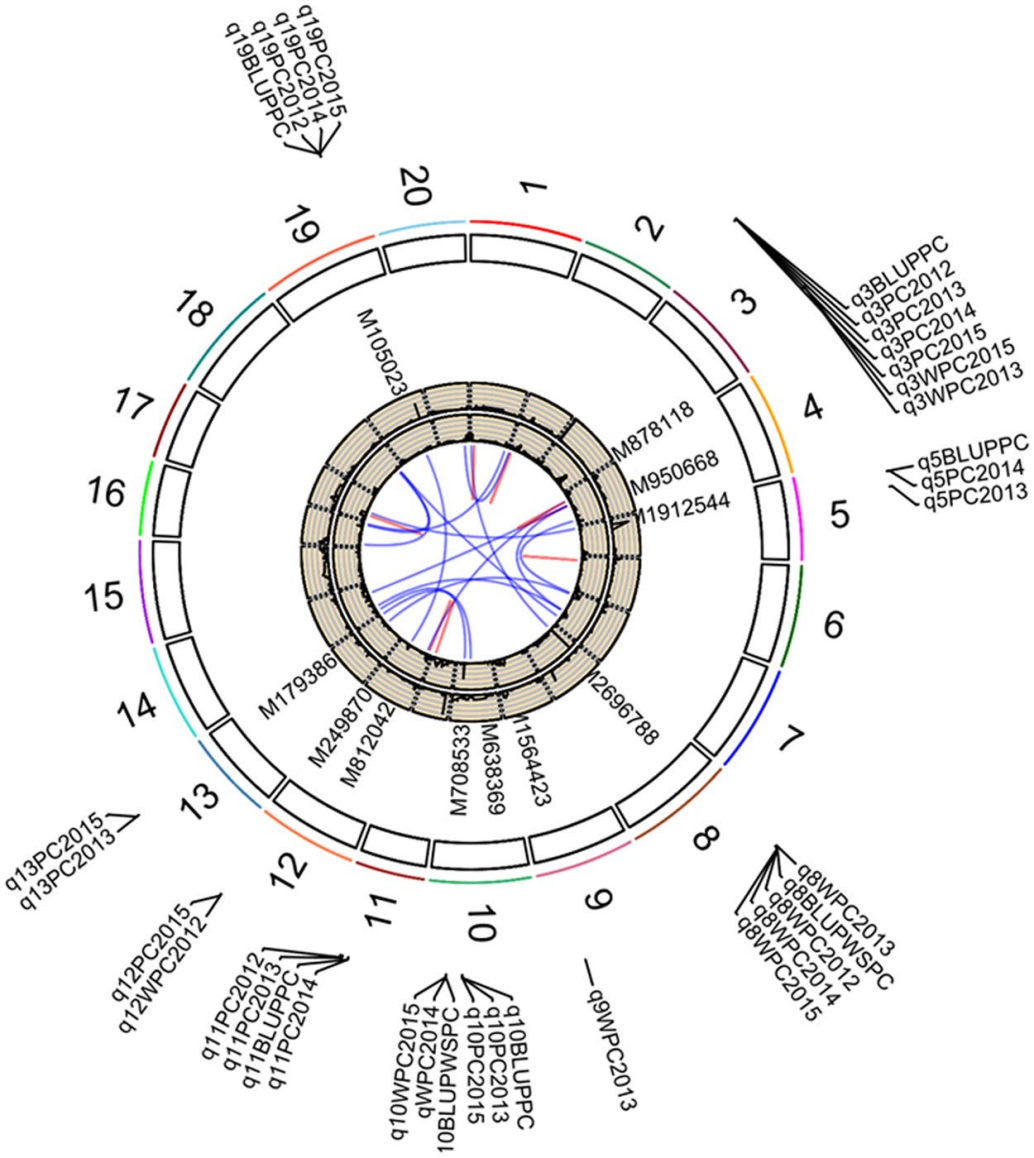
QTLs for soybean protein content (PC) and water-soluble protein content (WSPC) on soybean chromosomes by linkage mapping in RIL population. The lines link denotes epistatic associations between QTL and QTL. Blue line denotes two QTLs in different chromosomes, while red line denotes two QTLs in the same chromosome. The outside/inside wheat-colored circle indicates the LOD/PVE value curve for investigated traits across environments. The outermost circle indicates the 20 soybean chromosomes, QTLs for PC/WSPC, the position and linked markers of these QTLs on the chromosomes.

In this study, a complex genetic architecture underlying natural variation of soybean PC and WSPC was unraveled through GWAS. To validate the GWAS signals, we conducted QTL mapping for PC and WSPC using 152 F_8: 12_ RILs, with parents being previously selected from the GWAS association panel. Based on the high-density genetic map (6,159 SNPs) (Zhang *et al*.^15^), six and three QTLs underlying soybean PC and WSPC, respectively, were identified across at least two environments (Table 2 and Fig. 5). Detailed information about these additive and epistatic QTLs across five environments and their BLUP was summarized in Tables S5 and S6. Of the nine QTLs identified via linkage mapping, six (*qPC3*, *qPC5*, *qPC10*, *qPC19*, *qWSPC8*, and *qWSPC10*) were co-located with the significant loci that were identified in GWAS. The high consistency between QTLs and GWAS results suggest the robustness of both mapping strategies, and further verify that the populations and methods for analyzing used here were appropriate for PC and WSPC analysis.

**Table 2:** Nine QTLs associated with protein content (PC) and water-soluble protein content (WSPC) across four environments in RIL population.

Traits	Name^a^	Chr.^b^	Marker interval^c^	Position^d^	LOD^e^					R^2^(%)^f^	Add^g^
					2012	2013	2014	2015	BLUP		
PC	***qPC3***	3	M950668-M977935	38993729–39764723	5.54	3.82	5.8	2.6	7.28	11.67	−0.46
	*qPC5*	5	M1912544-M1913462	5517334–5608744	ns	3.47	2.7	ns	3.32	6.87	0.36
	*qPC9*	9	M1614684-M1595567	45450837–45451129	2.75	ns	2.60	ns	2.63	7.24	0.36
	*qPC10*	10	M764990-M697081	37059790–37175436	ns	3.09	ns	5.23	4.11	9.1	0.61
	*qPC13*	13	M1793867-M1714063	34799842–35437945	ns	3.45	ns	2.76	ns	6.34	0.36
	*qPC11*-*1*	11	M812042-M837694	7962054–8577956	2.76	2.81	3.16	ns	3.99	6.74	−0.36
	*qPC11* - *2*	11	M804544-M861597	7301888–7428451	6.27	3.70	6.45	4.04	6.95	17.47	−0.55
	*qPC19*	19	M1050238-M1066605	47835501–49307483	3.3	ns	3.35	2.83	2.86	6.58	−0.35
WSPC	*qWSPC1*	1	M430744-M433677	294462–294750	ns	3.93	ns	ns	4.15	9.53	0.72
	***qWSPC3***	3	M878118-M941301	1452355–1990434	ns	3.27	ns	2.5	ns	6.53	0.7
	***qWSPC8***	8	M2696788-M2699628	8709744–9076486	3	4.28	7.96	8.65	9.04	17.93	1.04
	*qWSPC10*	10	M812042-M837694	38151603–38151909	ns	ns	2.62	2.98	2.87	6.94	0.48

^a^The name of the QTL is defined by the abbreviation of traits and the chromosome number. ^b^Chromosome; ^c^confidence interval of QTL; ^d^The interval of physical distance in soybean genome; ^e^the logarithm of odds score; ^f^the mean phenotypic variance explained by related QTL; ^g^the mean additive effect of QTLs. The QTLs in bold were detected simultaneously by ICIM and GCIM, and the underlined QTLs were detected by GCIM only.

To increase the statistical power in the detection of small-effect and linked QTLs, controlling background via selecting markers in the CIM was replaced by estimating polygenic variance in GWAS. This method was named genome-wide composite interval mapping (GCIM). The GCIM was also used to analyze the above datasets. As a result, major QTLs, detected by ICIM, could still be identified in various environments (Tables 2 and S5). For example, the QTL on Gm08 could still be detected in all the environments and their BLUP. Meanwhile, additional loci were found by the GCIM. For instance, a novel QTL, *qPC9*, on Gm09 was detected in three environments and their BLUP.

### Genetic relationship between PC and WSPC

To evaluate the genetic relationship between PC and WSPC, we compared the mapping results for both traits. In GWAS results, four of 32 genomic regions (16 for PC and 16 for WSPC) exhibited pleiotropic effects for both PC and WSPC (Table 1 and Fig. 2), suggesting that PC and WSPC are commonly regulated at a moderate level. It was consistent with the phenotype correlation between both traits (Fig. 1). For example, the SNP, AX-93933852, on Gm10 affecting soybean both PC and WSPC was identified across all six environments and explained 13.0–32.4% of the phenotypic variation. The other significant SNP, AX-93795201, on Gm11 was detected for both PC and WSPC across five environments and the average -log *P* of this loci was 6.94 (ranged from 5.6 to 8.6). The third region containing the SNP, AX-94196006, on Gm19 was associated with both PC and WSPC across all six environments and explained 7.9–26.7% of the phenotypic variation. These four loci on Gm10, 11, 12, and 19 (Table 1) explained high phenotypic variance and were genetically stable across years, suggesting that these genomic regions play an important role in contributing to the variation of PC and WSPC.

**Figure 6:**
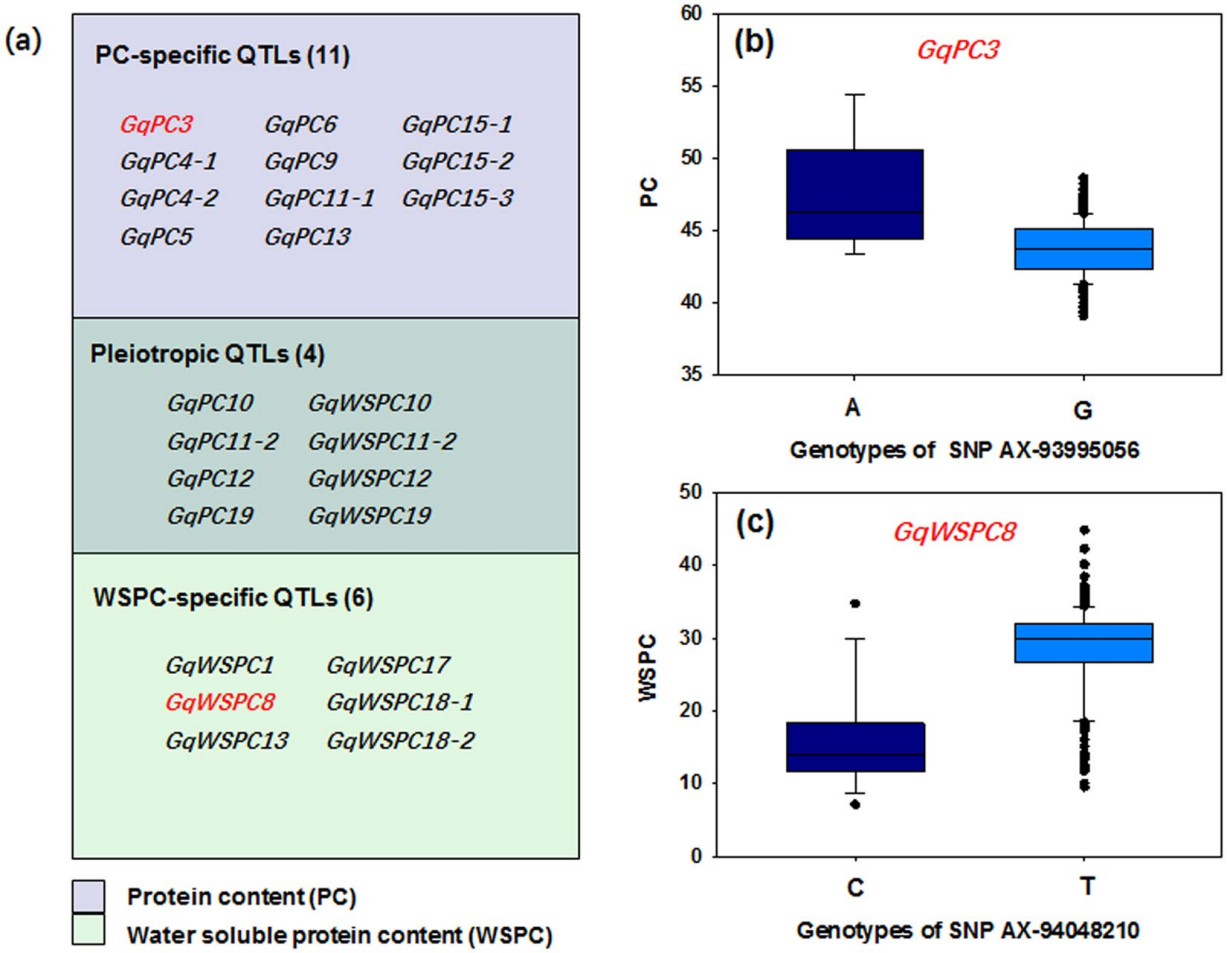
The genetic overlap between protein content (PC) and water-soluble protein content (WSPC) in the GWAS population. (**a**) Quantitative trait locus (QTL) categories and their number. (**b**) Associations between genotypes of SNP AX-93995056 and PC. Box plot of PC in 14 A-type and 195 G-type soybean accessions. The vertical axis indicates the PC. The PC of A-type accessions was significantly higher than that of G-type accessions (*t* test, *P* = 3.17 × 10^−8^). (**c**) Associations between genotypes of SNP AX-94048210 and WSPC. Box plot of PC in 50 A-type and 169 G-type soybean accessions. The vertical axis indicates the WSPC. The WSPC of G-type accessions was significantly higher than that of A-type accessions (*t* test, *P* = 1.01 × 10^−29^).

In addition, we also identified a total of 11 PC-specific loci on Gm03, 4, 5, 6, 9, 11, 13, and 15 across at least three environments, five of these loci are novel (Table 1 and Fig. 6a). Briefly, a major PC-specific locus, *GqPC3*, identified here co-localized with previous identified QTLs^38, 39^ and soybean storage protein-related genes, *G2* and *G3* ^9, 10^. Moreover, *GqPC3* was also identified by linkage mapping and explained 11.7% of the phenotypic variance (Table 2). A comparison of the PC of 196 G-type and 23 A-type soybean accessions genotyped at the leader AX-93995056 demonstrated that the PC in the accessions with A genotype at this locus was increased by 9.5% relative to the G-type accessions (*t* test, *P* = 3.17 × 10^−8^) (Fig. 6b).

Meanwhile, we also identified six loci specific for WSPC on Gm01, 8, 13, 17, and 18 across at least three environments, suggesting that these loci might be responsible for the low WSPC of soybean varieties with high PC. It is important to note that the WSPC-specific signal (around AX-94048210 on Gm08) that was identified across all environments by GWAS was also previously identified associated with soybean WSPC^8, 13^. This region affecting soybean WSPC was stably identified across environments and could explain 19.3% (ranged from 8.7 to 33.8%) of phenotypic variance, with an average increase of 38.2% of WSPC per T allele (Fig. 6c). These WSPC-specific loci might be important for the genetic variation in WSPC and could be used in the marker-assistant breeding of soybean with high WSPC.

For the QTLs underlying PC and WSPC in linkage mapping, only one QTL commonly responsible for PC and WSPC was detected. This overlap is not significantly different from expected by chance alone (*P* = 0.072), suggesting that PC and WSPC might be under relatively independent genetic control in the RIL population. This result is consistent with the lower phenotypic correlation between PC and WSPC when compared with the phenotypic correlation of WSPC/PC itself (Figs 1 and S1). In addition, the analysis of the positive or negative effect of QTL revealed that the alleles at three (50%) of six QTLs for PC were associated with the effect of decreasing the soybean protein content (Table 2). However, in contrast to the allelic effect distribution of PC QTLs, all the alleles of three QTLs for WSPC tended to increase the WSPC (Table 2). This contrasting QTL allele effect direction for PC and WSPC suggests that the two traits may be under differentially directional selection during soybean improvement.

### Candidate genes underlying the QTLs for PC and WSPC

In this study, the major loci for PC and WSPC identified via GWAS had considerable overlaps with previously reported QTLs related to soybean protein or its components, further supporting to our findings (Table 1). Of the 32 identified PC/WSPC loci, more than two-thirds (22/32) were located within the previously-found QTLs or overlapped with the many QTLs identified in the present study by linkage mapping (Table 1). Moreover, we also found that several of these QTLs contain previously reported genes related to soybean protein. For example, the locus (*GqPC3*) affecting soybean PC that was detected in both GWAS and linkage mapping localized at a genomic region ~40 kb away from a published glycinin gene, *Gy2* (encoding glycinin *A2B1a* precursor)^9, 10^. Another major QTL (*GqPC13*) on Gm13 associated with PC identified in this (Tables 1 and 2) and previous studies^40^ contain a glycinin gene (*Glyma*.*13G123500*) encoding glycinin *A3B4* subunits related soybean protein^41, 42^. The third overlap include a stably QTL (*GqPC19*) on Gm19 that was significantly associated with soybean PC and WSPC via association and linkage analysis in our (Table 1 and Fig. 2) and previous studies^8, 43^. This QTL contains a globulin gene (*Glyma*.*19G236600*) encoding 7S globulin precursor^44, 45^. The co-localization of previously reported protein-related QTLs/genes with QTLs identified here provideds a strong evidence showing the robustness of the mapping results.

Identification of candidate gene underlying QTL relies on high mapping resolution. However, the accuracy and precision of locating QTL depend, in part, on the density of molecular markers^46^. In this study, the average SNP spacing was approximately 3.3 kb along the 20 chromosomes of soybean (975 Mb)^16^, this resolution is much higher compared with the average SNP spacing of ~850 kb in a previous study^8^. Based on the high mapping resolution, candidate genes potentially underlying these main-effect loci (identified at least across three environments) associated with PC and WSPC could be predicted (Table 1). Moreover, examination of the expression pattern of these genes during the seed developmental stages would be helpful for us to understand its relevence to the traits (Table S7). We found that five of these genes were expressed specifically in seed developmental stages (Table S7), suggesting that these genes may be involved in the accumulation of soybean seed protein. Two candidate genes (*Glyma*.*13G123500* and *Glyma*.*13G194400*) that were located within the QTL significantly associated with both PC and WSPC were highly expressed in seeds, especially during late developmental stages. In addition, expression of three genes (*Glyma*.*15G098100*, *Glyma*.*08G112300*, and *Glyma*.*08G113400*) could also be detected in seeds, leaves, flowers, root and nodule. The roles of these genes potentially involved in the seeds protein composition need further determination.

In this study, we were particularly interested in the loci that can enhance the solubility of soybean protein or related to WSPC, such as the WSPC-specific SNP locus AX-94048210 (MAF = 0.15) on Gm08 that was repeatedly detected in both the present and previous studies^8^. In comparison with the alternative allele, the desired alleles of this locus may increase the soybean WSPC more than 30% (Fig. 6c). Further analysis showed that AX-94048210 tagged to an LD block harboring two candidate genes encoding seed storage 2S albumin protein (*Glyma*.*08G112300*) and amino acid transporter (*AAP8*, *Glyma*.*08G113400*), respectively (Fig. 3b,c), which may involve in the regulation of storage protein biosynthesis in soybean^47^.

Another important WSPC-specific QTL, *Gq18*-*1*, on Gm18 were detected across all environments, this QTL was previously identified to be associated with soybean protein^43^. In this region, *Glyma*.*18G071900* encoding an amino acid permease located under the peak SNP (AX-93870261) of *Gq18*-*1* was regarded as a promising candidate, as amino acid permease can improve plant nitrogen status and lead to higher seed protein contains by increasing seed sink strength for nitrogen^11, 47^.

For epistasis loci, although some of the SNP-SNP interactions included the SNPs that were not significant in GWAS (Fig. 4a), we found that the certain allels combinations showed a strong epistatic effect by EGWAS (Fig. 4b). For example, two SNPs, AX-93822697_T_A (MAF = 0.16) and AX-93952504 _G_T (MAF = 0.13), conferring the interaction between Gm14 and Gm18, were located within known protein related loci^48^. Further examination of the gene candidates for these loci revealed that the putative gene *Glyma*.*14G087900* containing AX-93822697_T_A on Gm14 and *Glyma*.*18G229300* containing AX-93952504 _G_T on Gm18 encode an aminotransferase and a malate enzymes, respectively (Fig. 4c,d). Aminotransferase (AAT) is a key enzyme in the synthesis of amino acids and involved in the regulation of carbon and nitrogen metabolism in almost all organisms. Over-expression of aspartate aminotransferase genes in *Arabidopsis* ^49^ or rice^50^ resulted in altered nitrogen metabolism and increased amino acid content in seeds. Malic enzymes (ME) catalyze the decarboxylation of malate generating pyruvate, CO_2_, and NADH or NADPH., ME also involved in oil biosynthesis in plants^51^, and is negatively correlated with protein synthesis and compete for the same basic substrates^52^. The roles of the two genes in the regulation of soybean protein merit further determination.

## Conclusions

We identified a total of 32 additive loci and 51 epistatic interactions associated with soybean PC and WSPC at various environments by applying the high-resolution GWAS mapping, demonstrating that epistatic effects are a substantial complement to additive effects in contributing to soybean protein. We also identified ten novel loci (*GqPC4*-*1*, *GqPC5*, *GqPC6*, *GqPC11*-*2*, *GqPC15*-*2*, *GqWSPC11*-*2*, *GqWSPC12*-*1*, *GqWSPC15*, *GqWSPC16* and *GqWSPC18*-*2*) associated with soybean PC and WSPC. Phenotypic correlation and QTL contrastive analysis exhibited a moderate level of genetic sharing and different genetic effect, suggesting that both traits tend to be under relatively independent genetic control and should be under differential directional selection during soybean improvement. These results provide important genetic insights into the high PC in soybean varieties with low WSPC and a genetic basis for improvement in soybean protein quality through marker-assisted selection and genomic selection. The putative amino acid transporter-encoding gene, *AAP8*, and other candidate genes potentially involved in soybean protein synthesis were the promising candidate genes meriting further determination. Further studies, such as expression profiling and functional analyses of these candidate genes, will be helpful to facilitate the elucidation of the molecular mechanisms underlying soybean protein content and its solubility.

## Materials and Methods

### Plant materials

The association panel for GWAS consisted of a diverse collection of 219 soybean accessions (including 195 landraces and 24 elite varieties) originated from 26 different provinces and six different ecological regions of soybean growing areas in China, ranging from latitude 53 to 24°N and longitude 134 to 97°E^16, 53^.

A segregating soybean population consisting of 152 F_8:12_ RILs that were derived from a cross between varieties “Nannong94-156” (male parent, low protein varieties) and “Bogao” (female parent, high protein varieties) was used to map QTL for soybean WSPC and PC. Both varieties were also included in the association panel, and a high-density genetic map containing 6,159 SNPs were used as previously described^24^.

### Field experiments and phenotyping

For the association panel, the field experiments were performed in 2009, 2011, 2012, 2013 and 2014 growing seasons at the following four different geographic locations: Jiangpu Experimental Station of Nanjing Agricultural University (32.1°N 118.4°E), Nanjing, in 2009 (designated as E1); Maozhuang Experimental Station of Henan Agricultural University (34.8°N 113.6°E), Zhengzhou, in 2009 (designated as E2) and 2011 (designated as E3); the Experimental Farm of Henan Agricultural University (33.2°N 112.9°E), Fangcheng, in 2012 (designated as E4), and Yuanyang Experimental Station of Henan Academy of Agricultural Sciences, Zhengzhou, in 2013 (designated as E5) and in 2014 (designated as E6). For the RIL population, field experiments were performed in 2012 (E4), 2013 (E5), 2014 (E6) and 2015 (same location as E6). A randomized block design was used for all field trials. All accessions were planted in two replications at E1 and in three replications at all other growing environments (E2, E3, E4, E5 and E6). In all environments, each accession was planted in three rows per plot, with each row was 200-cm long and 50-cm row spacing.

Measurement of soybean WSPC and PC was conducted using a near infrared spectrophotometer (NIR) seed analyzer (DA7200, Perten Instrument, Huddinge, Sweden) as previous described^8^. Briefly, approximately 60-g dry seeds per sample was fitted in a 75-mm-diameter cup, and the cup was rotated during NIRS scanning. The average value of three scans per sample were used in data analysis. These calibrations involved more than 700 uniform soybean samples that varied in seed PC, oil content, and 146 soybean samples in seed WSPC.

### Genotyping and genetic map

This association panel was genotyped using NJAU 355 K SoySNP array as previously described^16^, and a total of 292,035 high-quality single nucleotide polymorphism (SNPs) were used for association mapping. The number of SNPs is estimated to provide approximately one SNP per 3.3 kb along the 20 soybean chromosomes. In this study, SNPs with MAFs less than 5% were excluded from further analysis. The final set of 201,994 SNPs distributed over the whole soybean genome (20 chromosomes) was used to study genetic diversity, population structure, genetic relatedness and marker-trait associations in relation to genetic distance.

The linkage map used in this study was constructed as previously described^24^. This map, spanning 3020.59 cM in length, contained 6,159 SNP markers on 20 chromosomes, with an average distance of 0.49 cM between adjacent markers.

### Statistical analysis

Phenotypic data for soybean seed WSPC and PC across different environments were used to an ANOVA using the PROC GLM (general linear model) mixed model of SAS (version 9.2). The linear statistical model includes the effects of genotype, environment and the environment × genotype interaction. The decomposition of variance components was evaluated using PROC VARCOMP. The correlation coefficients between WSPC and PC in soybean were calculated with PROC CORR. The BLUP for each line was predicted with PROC MIXED in SAS and used as the phenotypic input in subsequent GWAS and QTL mapping.

The linkage disequilibrium (LD) block structure was examined using Hapview4.2 software by estimating the squared allele frequency correlation (*r* ^2^) of alleles in each QTL region. The significance of LD between the sites was determined using Fisher’s exact test.

### Genome-wide association mapping

SNPs with MAF ≥ 0.05 and the number of accessions with the minor allele ≥10 in the diverse panel were used to carry out GWAS. As a result, the final set of 201,994 SNPs (MAF ≥ 0.05) and the phenotypic values for all genotypes from the association panel across six environments and the BLUP over all environments, were used to perform marker-trait association analysis. GWAS was performed with a compressed mixed linear model^28^ using the GAPIT package^54^. The population structure was accounted for by principle component analysis (PCA) and the relatedness was calculated by VanRaden method^55^ using GAPIT. Markers were identified as significantly associated with traits by comparison with the significant threshold of P-value < 1/n (*P* < 4.95 × 10^−6^)^56^.

The genome-wide epistatic interaction test was implemented in PLINK version 1.07^57^. To remedy the shortcoming of a less stringent significance threshold (1/*n*), we also carried out multi-locus GWAS by using ISIS EM-BLASSO^58^ and mrMLM^59^ (with the software rmMLM: https://cran.r-project.org/web/packages/mrMLM/index.html) simultaneously in natural population.

### Linkage QTL mapping

For the RIL population, measurement of WSPC and PC was conducted using the same method as described above. The additive and epistatic QTLs underlying the WSPC and PC were identified by the QTL IciMapping program v4.0^60^ using single environment phenotypic values and the best linear unbiased prediction (BLUP) over all environments. Briefly, for the additive QTL, the inclusive composite interval mapping (ICIM) method was used in the software, the *P*-values for entering variables (PIN) and removing variables (POUT) were set at 0.01 and 0.02, and the scanning step was 2 cM. The ICIM-EPI method was used to detect epistatic QTL, the PIN and POUT were set at 0.0001 and 0.0002, respectively. The LOD thresholds for each index of QTL were determined by 1000 permutation test at 95% confidence level. In addition, to evaluate the significance of correspondence of QTLs underlying soybean PC and WSPC, a previously statistical test based on the hypergeometric probability function was used to calculate the probability of QTL correspondence by chance alone as described by Lin *et al*.^61^. Identification of small-effect and linked QTLs were performed using GCIM as previously described^62^.

### Prediction of candidate genes

To uncover the candidate genes underlying association signals, the predicted genes in the target genomic regions were retrieved from the annotation of the soybean reference genome (Wm82.a2.v1) in Phytozome v10.3 (http://phytozome.net) and were manually analyzed using protein blasting (http://www.ebi.ac.uk/Tools/sss/ncbiblast/). First, we selected the candidate genes in the region defined by clustering of trait-associated SNPs at LD *r* ^*2*^ > 0.70 or in a region of 70 kb each side of the peak SNP. Genes with known functional descriptions related to soybean protein content or participating in seed protein synthesis pathway were selected as candidate genes. The expression data of these candidate genes at soybean seed developmental stages were retrieved from Phytozome (https://phytozome.jgi.doe.gov/) and Soybase (https://soybase.org/ soyseq/) to analysis its relevance to PC and/or WSPC.

## Electronic supplementary material

Supplemental Information
