# Supplementary material for: The genetic architecture of water-soluble protein content and its genetic relationship to total protein content in soybean
Source: Sci Rep. 2017 Jul 11;7:5053. doi: 10.1038/s41598-017-04685-7 (PMC5506034; doi:10.1038/s41598-017-04685-7)
Supplement: Supplementary file 1 — Supplemental Information [file 41598_2017_4685_MOESM1_ESM.doc]

**Supplemental Information**

**The genetic architecture of** **water-soluble protein content and its genetic relationship to total protein content in soybean**

**Dan Zhang****^1*^, Haiyan Lü^1^****^*^, Shanshan Chu^1^, Huairen Zhang^2^, Hengyou Zhang^3^, Yuming Yang^4^, Hongyan Li****^1^ & Deyue Yu^4^**

^1^Collaborative Innovation Center of Henan Grain Crops, College of Agronomy, Henan Agricultural University, Zhengzhou 450002, China; ^2^ The Institute of Genetics and Developmental Biology (IGDB) of the Chinese Academy of Sciences, Beijing, 100101, China; ^3^ Department of Biological Sciences, University of North Carolina at Charlotte, Charlotte, NC 28223, USA; ^4^ National Center for Soybean Improvement, National Key Laboratory of Crop Genetics and Germplasm Enhancement, Nanjing Agricultural University, Nanjing 210095, China.

** These authors contributed equally to this work.* Correspondence and requests for materials should be addressed to D. Z. (email: zhangd@henau.edu.cn) and D.Y. (email: dyyu@njau.edu.cn)

Tel.: +86-371-63555076

Fax: +86-371-63555076

**
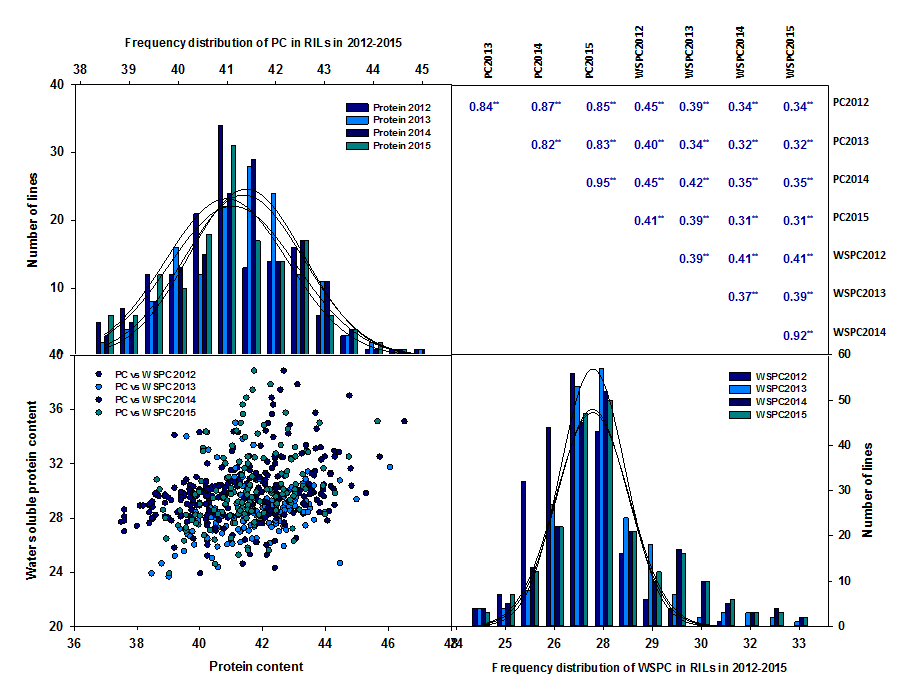
**

**Figure S1. Phenotypic analysis of protein content (PC) and water-soluble protein content (WSPC) in the 152 recombinant inbred lines.** The histograms on the diagonal show the phenotypic distribution of each trait. The values above the diagonal are pairwise correlation coefﬁcients between traits, and the plots below the diagonal are scatter plots of compared traits. PCE2012-2015, denote the field experiments were performed in 2012-2015; WSPC2012-2015, denote the water-soluble protein content in the corresponding years.


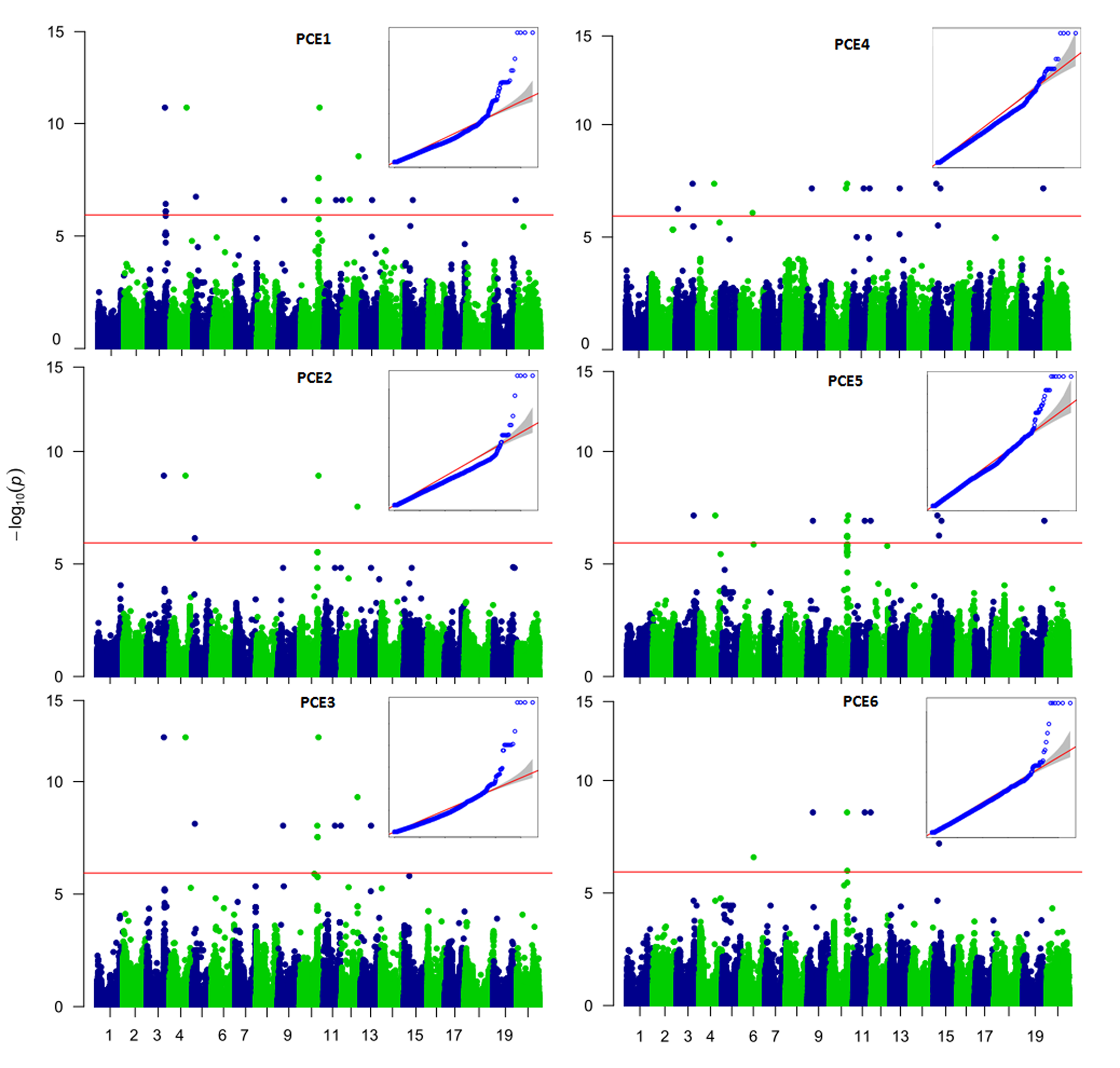


**Figure S2. Genome-wide association study (GWAS) for protein content (PC) across six environments in soybean.** Manhattan plots with the matching small QQ plots are shown in the same ﬁgure for PC in different environments. Red horizontal lines depict the Bonferroni-adjusted significance threshold (*P* < 4.95 × 10^−6^).


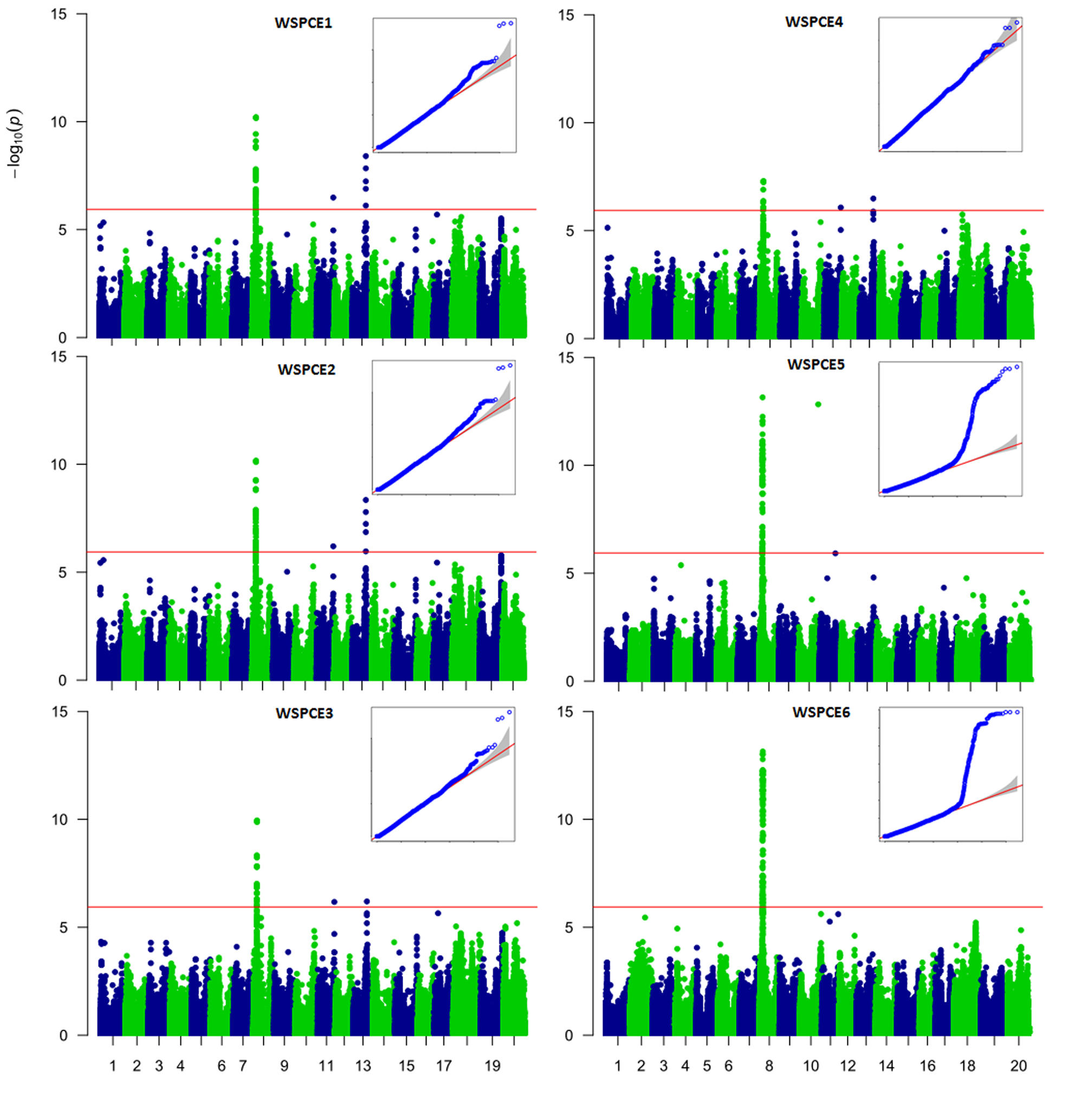


**Figure S3. Genome-wide association study (GWAS) for water-soluble protein content (WSPC) across six environments in soybean.** Manhattan plots with the matching small QQ plots are shown in the same ﬁgure for WSPC in different environments. Red horizontal lines depict the Bonferroni-adjusted significance threshold (*P* < 4.95 × 10^−6^).

Table S1 The means, standard deviation, range and skew of soybean protein content (PC) and water soluble protein content (WSPC) in natural population (214 soybean accessions) and RIL population (152 recombinant inbred lines)

| **Populations** | **Traits** | **Mean (%)** | **SD (%)** | **Min (%)** | **Max (%)** | **Skew** | **Kurt** | **G^a^** | **E^b^** | **GⅹE^c^** | ***h*^2 d^** |
| --- | --- | --- | --- | --- | --- | --- | --- | --- | --- | --- | --- |
| **GWAS population** | PC-E1 | 39.97 | 4.27 | 30.27 | 54.86 | 0.34 | 0.4 | ** | ** | ** | 40.20% |
|  | PC-E2 | 44.69 | 3.51 | 36.19 | 53.07 | 0.04 | -0.63 |  |  |  |  |
|  | PC-E3 | 41.92 | 3.34 | 34.66 | 54.68 | 0.34 | 0.53 |  |  |  |  |
|  | PC-E4 | 45.6 | 1.87 | 41.32 | 52 | 0.4 | 0.39 |  |  |  |  |
|  | PC-E5 | 45.87 | 2.47 | 40.67 | 57.12 | 0.55 | 1.27 |  |  |  |  |
|  | PC-E6 | 44.51 | 2.54 | 38 | 55 | 0.48 | 0.85 |  |  |  |  |
|  | WSPC-E1 | 24.89 | 8.42 | 6.32 | 45.43 | -0.61 | 0.08 | ** | ** | ** | 52.70% |
|  | WSPC-E2 | 27.91 | 7.67 | 7.41 | 45 | -1.02 | 0.67 |  |  |  |  |
|  | WSPC-E3 | 26.52 | 7.99 | 7.39 | 46.84 | -0.75 | 0.42 |  |  |  |  |
|  | WSPC-E4 | 29.29 | 7.62 | 9.3 | 45.99 | -1.09 | 0.7 |  |  |  |  |
|  | WSPC-E5 | 28.23 | 7.57 | 7.42 | 43.08 | -1.12 | 0.42 |  |  |  |  |
|  | WSPC-E6 | 27.73 | 7.11 | 8.69 | 41.24 | -1.17 | 0.97 |  |  |  |  |
| **RIL population** | PC2012 | 40.26 | 1.32 | 37.5 | 44.3 | 0.18 | 0.02 | ** | ** | ** | 61.10% |
|  | PC2013 | 42.35 | 1.45 | 37.6 | 46.52 | -0.16 | 0.58 |  |  |  |  |
|  | PC2014 | 42.4 | 1.35 | 39.2 | 46.52 | 0.09 | -0.01 |  |  |  |  |
|  | PC2015 | 42.08 | 1.48 | 37.43 | 46.46 | -0.08 | 0.56 |  |  |  |  |
|  | WSPC2012 | 29.6 | 1.53 | 25.2 | 34.3 | 0 | 0.94 | ** | ** | ** | 52.40% |
|  | WSPC2013 | 28.4 | 2.01 | 22.65 | 35.43 | 0.5 | 1.76 |  |  |  |  |
|  | WSPC2014 | 30.15 | 2.82 | 23.9 | 38.84 | 0.69 | 0.58 |  |  |  |  |
|  | WSPC2015 | 30.15 | 2.77 | 23.9 | 38.84 | 0.7 | 0.55 |  |  |  |  |

^a^ genotype; ^b^ environments; ^c^ genotype × environments, ^d^ broad-sense heritability

Table S2 The detailed significant SNP list for soybean protein content (PC) across six environments and the BLUP by GWAS

| **Methods** | **Traits** | **SNP** | **Chr.** | **Position** | **P.value/LOD score** | ***R*^2^** |
| --- | --- | --- | --- | --- | --- | --- |
| GAPIT | PCE1 | AX-93994414 | 3 | 35,995,285 | 1.95379E-11 | 0.330 |
| GAPIT | PCE2 | AX-93994414 | 3 | 35,995,285 | 1.18712E-09 | 0.258 |
| GAPIT | PCE3 | AX-93994414 | 3 | 35,995,285 | 1.07188E-12 | 0.352 |
| GAPIT | PCE4 | AX-93994414 | 3 | 35,995,285 | 4.21913E-08 | 0.272 |
| GAPIT | PCE5 | AX-93994414 | 3 | 35,995,285 | 7.00E-08 | 0.364 |
| GAPIT | PCBLUP | AX-93994414 | 3 | 35,995,285 | 5.56E-09 | 0.258 |
| GAPIT | PCE1 | AX-93995056 | 3 | 37,720,115 | 7.98746E-07 | 0.199 |
| GAPIT | PCE1 | AX-93995056 | 3 | 37,720,115 | 7.98746E-07 | 0.199 |
| GAPIT | PCE3 | AX-93995056 | 3 | 37,720,115 | 6.85608E-06 | 0.149 |
| GAPIT | PCE3 | AX-93995056 | 3 | 37,720,115 | 6.85608E-06 | 0.149 |
| GAPIT | PCE4 | AX-93995056 | 3 | 37,720,115 | 3.31108E-06 | 0.151 |
| GAPIT | PCE4 | AX-93995056 | 3 | 37,720,115 | 3.31108E-06 | 0.151 |
| GAPIT | PCBLUP | AX-93995056 | 3 | 37,720,115 | 2.89E-06 | 0.190 |
| GAPIT | PCE1 | AX-94274580 | 4 | 34,743,951 | 1.95379E-11 | 0.330 |
| GAPIT | PCE2 | AX-94274580 | 4 | 34,743,951 | 1.18712E-09 | 0.258 |
| GAPIT | PCE3 | AX-94274580 | 4 | 34,743,951 | 1.07188E-12 | 0.352 |
| GAPIT | PCE4 | AX-94274580 | 4 | 34,743,951 | 4.21913E-08 | 0.272 |
| GAPIT | PCE5 | AX-94274580 | 4 | 34,743,951 | 7.00E-08 | 0.364 |
| GAPIT | PCE6 | AX-94274580 | 4 | 34,743,951 | 2.20002E-05 | 0.210 |
| GAPIT | PCBLUP | AX-94274580 | 4 | 34,743,951 | 5.56E-09 | 0.258 |
| GAPIT | PCE3 | AX-93920058 | 4 | 46,200,673 | 5.26614E-06 | 0.152 |
| GAPIT | PCE4 | AX-93920058 | 4 | 46,200,673 | 2.22877E-06 | 0.213 |
| GAPIT | PCE5 | AX-93920058 | 4 | 46,200,673 | 3.63E-06 | 0.329 |
| GAPIT | PCE6 | AX-93920058 | 4 | 46,200,673 | 1.71961E-05 | 0.212 |
| GAPIT | PCE1 | AX-94012201 | 5 | 5,637,601 | 1.78811E-07 | 0.176 |
| GAPIT | PCE2 | AX-94012201 | 5 | 5,637,601 | 7.10237E-07 | 0.177 |
| GAPIT | PCE3 | AX-94012201 | 5 | 5,637,601 | 7.43977E-09 | 0.203 |
| GAPIT | PCBLUP | AX-94012201 | 5 | 5,637,601 | 1.83E-06 | 0.139 |
| GAPIT | PCE4 | AX-93731783 | 6 | 26,098,086 | 8.33745E-07 | 0.213 |
| GAPIT | PCE5 | AX-93731783 | 6 | 26,098,086 | 1.35E-06 | 0.338 |
| GAPIT | PCE6 | AX-93731783 | 6 | 26,098,086 | 2.55673E-07 | 0.255 |
| GAPIT | PCE1 | AX-93764734 | 9 | 11,138,977 | 2.5173E-07 | 0.212 |
| GAPIT | PCE3 | AX-93764734 | 9 | 11,138,977 | 9.05972E-09 | 0.230 |
| GAPIT | PCE4 | AX-93764734 | 9 | 11,138,977 | 6.84176E-08 | 0.216 |
| GAPIT | PCE5 | AX-93764734 | 9 | 11,138,977 | 1.20E-07 | 0.359 |
| GAPIT | PCE6 | AX-93764734 | 9 | 11,138,977 | 2.60574E-09 | 0.305 |
| GAPIT | PCBLUP | AX-93764734 | 9 | 11,138,977 | 1.21E-07 | 0.224 |
| GAPIT | PCE1 | AX-93933535 | 10 | 37,843,671 | 2.5173E-07 | 0.212 |
| GAPIT | PCE3 | AX-93933535 | 10 | 37,843,671 | 9.05972E-09 | 0.230 |
| GAPIT | PCE4 | AX-93933535 | 10 | 37,843,671 | 6.84176E-08 | 0.216 |
| GAPIT | PCE5 | AX-93933535 | 10 | 37,843,671 | 1.20E-07 | 0.359 |
| GAPIT | PCE6 | AX-93933535 | 10 | 37,843,671 | 2.60574E-09 | 0.305 |
| GAPIT | PCBLUP | AX-93933535 | 10 | 37,843,671 | 1.21E-07 | 0.224 |
| GAPIT | PCE1 | AX-94077990 | 10 | 38,184,512 | 2.65809E-08 | 0.239 |
| GAPIT | PCE2 | AX-94077990 | 10 | 38,184,512 | 2.98939E-06 | 0.160 |
| GAPIT | PCE3 | AX-94077990 | 10 | 38,184,512 | 2.90832E-08 | 0.215 |
| GAPIT | PCE5 | AX-94077990 | 10 | 38,184,512 | 6.19E-07 | 0.345 |
| GAPIT | PCBLUP | AX-94077990 | 10 | 38,184,512 | 2.10E-06 | 0.193 |
| GAPIT | PCE1 | AX-93781448 | 10 | 38,413,061 | 2.71523E-07 | 0.211 |
| GAPIT | PCE3 | AX-93781448 | 10 | 38,413,061 | 1.72086E-06 | 0.166 |
| GAPIT | PCE5 | AX-93781448 | 10 | 38,413,061 | 1.36E-06 | 0.338 |
| GAPIT | PCE1 | AX-94078083 | 10 | 38,417,606 | 2.71523E-07 | 0.211 |
| GAPIT | PCE3 | AX-94078083 | 10 | 38,417,606 | 1.72086E-06 | 0.166 |
| GAPIT | PCE5 | AX-94078083 | 10 | 38,417,606 | 1.36E-06 | 0.338 |
| GAPIT | PCE1 | AX-94078084 | 10 | 38,419,798 | 2.65809E-08 | 0.239 |
| GAPIT | PCE2 | AX-94078084 | 10 | 38,419,798 | 2.98939E-06 | 0.160 |
| GAPIT | PCE3 | AX-94078084 | 10 | 38,419,798 | 2.90832E-08 | 0.215 |
| GAPIT | PCE5 | AX-94078084 | 10 | 38,419,798 | 6.19E-07 | 0.345 |
| GAPIT | PCBLUP | AX-94078084 | 10 | 38,419,798 | 2.10E-06 | 0.193 |
| GAPIT | PCE1 | AX-93933852 | 10 | 40,304,375 | 1.95379E-11 | 0.330 |
| GAPIT | PCE2 | AX-93933852 | 10 | 40,304,375 | 1.18712E-09 | 0.258 |
| GAPIT | PCE3 | AX-93933852 | 10 | 40,304,375 | 1.07188E-12 | 0.352 |
| GAPIT | PCE4 | AX-93933852 | 10 | 40,304,375 | 4.21913E-08 | 0.272 |
| GAPIT | PCE5 | AX-93933852 | 10 | 40,304,375 | 7.00E-08 | 0.364 |
| GAPIT | PCBLUP | AX-93933852 | 10 | 40,304,375 | 5.56E-09 | 0.258 |
| GAPIT | PCE1 | AX-94089116 | 11 | 24,782,059 | 2.5173E-07 | 0.212 |
| GAPIT | PCE3 | AX-94089116 | 11 | 24,782,059 | 9.05972E-09 | 0.230 |
| GAPIT | PCE4 | AX-94089116 | 11 | 24,782,059 | 6.84176E-08 | 0.216 |
| GAPIT | PCE5 | AX-94089116 | 11 | 24,782,059 | 1.20E-07 | 0.359 |
| GAPIT | PCE6 | AX-94089116 | 11 | 24,782,059 | 2.60574E-09 | 0.305 |
| GAPIT | PCBLUP | AX-94089116 | 11 | 24,782,059 | 1.21E-07 | 0.224 |
| GAPIT | PCE1 | AX-93795201 | 11 | 37,461,551 | 2.5173E-07 | 0.212 |
| GAPIT | PCE3 | AX-93795201 | 11 | 37,461,551 | 9.05972E-09 | 0.230 |
| GAPIT | PCE4 | AX-93795201 | 11 | 37,461,551 | 6.84176E-08 | 0.216 |
| GAPIT | PCE5 | AX-93795201 | 11 | 37,461,551 | 1.20E-07 | 0.359 |
| GAPIT | PCE6 | AX-93795201 | 11 | 37,461,551 | 2.60574E-09 | 0.305 |
| GAPIT | PCBLUP | AX-93795201 | 11 | 37,461,551 | 1.21E-07 | 0.224 |
| GAPIT | PCE1 | AX-93804391 | 12 | 34,037,114 | 2.83876E-09 | 0.267 |
| GAPIT | PCE2 | AX-93804391 | 12 | 34,037,114 | 2.81239E-08 | 0.217 |
| GAPIT | PCE3 | AX-93804391 | 12 | 34,037,114 | 4.89097E-10 | 0.268 |
| GAPIT | PCE5 | AX-93804391 | 12 | 34,037,114 | 1.57E-06 | 0.337 |
| GAPIT | PCBLUP | AX-93804391 | 12 | 34,037,114 | 5.66E-07 | 0.207 |
| GAPIT | PCE1 | AX-94109235 | 13 | 23,091,289 | 2.5173E-07 | 0.212 |
| GAPIT | PCE3 | AX-94109235 | 13 | 23,091,289 | 9.05972E-09 | 0.230 |
| GAPIT | PCE4 | AX-94109235 | 13 | 23,091,289 | 6.84176E-08 | 0.216 |
| GAPIT | PCE1 | AX-93649790 | 15 | 7,681,400 | 1.95379E-11 | 0.330 |
| GAPIT | PCE2 | AX-93649790 | 15 | 7,681,400 | 1.18712E-09 | 0.258 |
| GAPIT | PCE3 | AX-93649790 | 15 | 7,681,400 | 1.07188E-12 | 0.352 |
| GAPIT | PCE4 | AX-93649790 | 15 | 7,681,400 | 4.21913E-08 | 0.272 |
| GAPIT | PCE5 | AX-93649790 | 15 | 7,681,400 | 7.00E-08 | 0.364 |
| GAPIT | PCE6 | AX-93649790 | 15 | 7,681,400 | 2.20002E-05 | 0.210 |
| GAPIT | PCBLUP | AX-93649790 | 15 | 7,681,400 | 5.56E-09 | 0.258 |
| GAPIT | PCE1 | AX-93837099 | 15 | 11,235,816 | 3.58349E-06 | 0.182 |
| GAPIT | PCE3 | AX-93837099 | 15 | 11,235,816 | 1.55095E-06 | 0.167 |
| GAPIT | PCE4 | AX-93837099 | 15 | 11,235,816 | 3.03195E-06 | 0.194 |
| GAPIT | PCE5 | AX-93837099 | 15 | 11,235,816 | 5.43E-07 | 0.346 |
| GAPIT | PCE6 | AX-93837099 | 15 | 11,235,816 | 6.32979E-08 | 0.270 |
| GAPIT | PCBLUP | AX-93837099 | 15 | 11,235,816 | 1.14E-06 | 0.200 |
| GAPIT | PCE1 | AX-94136114 | 15 | 16,417,543 | 2.5173E-07 | 0.212 |
| GAPIT | PCE3 | AX-94136114 | 15 | 16,417,543 | 9.05972E-09 | 0.230 |
| GAPIT | PCE4 | AX-94136114 | 15 | 16,417,543 | 6.84176E-08 | 0.216 |
| GAPIT | PCE5 | AX-94136114 | 15 | 16,417,543 | 1.20E-07 | 0.359 |
| GAPIT | PCE6 | AX-94136114 | 15 | 16,417,543 | 2.60574E-09 | 0.305 |
| GAPIT | PCBLUP | AX-94136114 | 15 | 16,417,543 | 1.21E-07 | 0.224 |
| GAPIT | PCE1 | AX-93897264 | 19 | 45,130,762 | 2.5173E-07 | 0.212 |
| GAPIT | PCE3 | AX-93897264 | 19 | 45,130,762 | 9.05972E-09 | 0.230 |
| GAPIT | PCE4 | AX-93897264 | 19 | 45,130,762 | 6.84176E-08 | 0.216 |
| GAPIT | PCE5 | AX-93897264 | 19 | 45,130,762 | 1.20E-07 | 0.359 |
| GAPIT | PCE6 | AX-93897264 | 19 | 45,130,762 | 2.60574E-09 | 0.305 |
| GAPIT | PCBLUP | AX-93897264 | 19 | 45,130,762 | 1.21E-07 | 0.224 |
| mrMLM | PCE1 | AX-93995057 | 3 | 37,675,520 | 5.728 | 0.136 |
| mrMLM | PCE3 | AX-93995057 | 3 | 37,675,520 | 4.416 | 0.068 |
| mrMLM | PCE5 | AX-93995057 | 3 | 37,675,520 | 5.021 | 0.121 |
| mrMLM | PCBLUP | AX-93995057 | 3 | 37,675,520 | 7.416 | 0.083 |
| mrMLM | PCE3 | AX-93748464 | 7 | 43,807,170 | 4.767 | 0.062 |
| mrMLM | PCBLUP | AX-93748464 | 7 | 43,807,170 | 3.628 | 0.061 |
| mrMLM | PCE5 | AX-94143896 | 16 | 3,021,765 | 4.676 | 0.033 |
| mrMLM | PCE6 | AX-94143896 | 16 | 3,021,765 | 5.941 | 0.088 |
| mrMLM | PCE1 | AX-94289790 | 17 | 39,096,765 | 4.688 | 0.088 |
| mrMLM | PCBLUP | AX-94289790 | 17 | 39,096,765 | 3.964 | 0.091 |
| ISIS EM-BLASSO | PCE5 | AX-93623103 | 4 | 3,331,813 | 7.788 | 0.053 |
| ISIS EM-BLASSO | PCE6 | AX-93623103 | 4 | 3,331,813 | 2.970 | 0.020 |
| ISIS EM-BLASSO | PCE3 | AX-94007216 | 4 | 41,012,908 | 6.576 | 0.040 |
| ISIS EM-BLASSO | PCBLUP | AX-94007216 | 4 | 41,012,908 | 7.316 | 0.053 |
| ISIS EM-BLASSO | PCE3 | AX-94045715 | 8 | 1,705,651 | 10.261 | 0.094 |
| ISIS EM-BLASSO | PCE6 | AX-94045715 | 8 | 1,705,651 | 6.734 | 0.039 |
| ISIS EM-BLASSO | PCE1 | AX-94060317 | 9 | 7,972,966 | 5.914 | 0.037 |
| ISIS EM-BLASSO | PCBLUP | AX-94060317 | 9 | 7,972,966 | 3.008 | 0.018 |
| ISIS EM-BLASSO | PCE1 | AX-93794111 | 11 | 33,343,425 | 6.724 | 0.046 |
| ISIS EM-BLASSO | PCBLUP | AX-93794111 | 11 | 33,343,425 | 2.847 | 0.012 |
| ISIS EM-BLASSO | PCE1 | AX-93822697 | 14 | 7,715,347 | 5.785 | 0.039 |
| ISIS EM-BLASSO | PCE2 | AX-93822697 | 14 | 7,715,347 | 3.440 | 0.015 |
| ISIS EM-BLASSO | PCE6 | AX-93822697 | 14 | 7,715,347 | 7.356 | 0.042 |
| ISIS EM-BLASSO | PCE5 | AX-93848616 | 16 | 7,791,494 | 3.697 | 0.017 |
| ISIS EM-BLASSO | PCE6 | AX-93848616 | 16 | 7,791,494 | 5.558 | 0.036 |
| ISIS EM-BLASSO | PCE5 | AX-94143896 | 16 | 3,021,765 | 4.676 | 0.033 |
| ISIS EM-BLASSO | PCE6 | AX-94143896 | 16 | 3,021,765 | 5.941 | 0.088 |
| ISIS EM-BLASSO | PCE3 | AX-94289608 | 17 | 29,961,204 | 3.140 | 0.041 |
| ISIS EM-BLASSO | PCE6 | AX-94289608 | 17 | 29,961,204 | 5.640 | 0.066 |
| ISIS EM-BLASSO | PCE1 | AX-94289790 | 17 | 39,096,765 | 4.688 | 0.088 |
| ISIS EM-BLASSO | PCBLUP | AX-94289790 | 17 | 39,096,765 | 3.964 | 0.091 |
| ISIS EM-BLASSO | PCE1 | AX-94178919 | 18 | 52,648,601 | 5.045 | 0.026 |
| ISIS EM-BLASSO | PCE6 | AX-94178919 | 18 | 52,648,601 | 3.671 | 0.015 |

Table S3 The detailed significant SNP list for soybean water soluble protein content (WSPC) across six environments and the BLUP by GWAS

| **Methods** | **Traits** | **SNP** | **Chr.** | **Position** | **P.value**  **LOD score** | ***R*^2^** |
| --- | --- | --- | --- | --- | --- | --- |
| GAPIT | WSPCE1 | AX-93964183 | 1 | 9,078,593 | 4.67E-06 | 0.098 |
| GAPIT | WSPCE2 | AX-93964183 | 1 | 9,078,593 | 2.75E-06 | 0.103 |
| GAPIT | WSPCE4 | AX-93964184 | 1 | 9,078,593 | 4.74E-06 | 0.085 |
| GAPIT | WSPCE1 | AX-93930655 | 8 | 8,013,021 | 4.56E-06 | 0.095 |
| GAPIT | WSPCE2 | AX-93930655 | 8 | 8,013,021 | 2.87E-06 | 0.099 |
| GAPIT | WSPCE5 | AX-93930655 | 8 | 8,013,021 | 6.04E-09 | 0.205 |
| GAPIT | WSPCE6 | AX-93930655 | 8 | 8,013,021 | 7.48E-10 | 0.243 |
| GAPIT | WSPCBLUP | AX-93930655 | 8 | 8,013,021 | 1.28E-07 | 0.299 |
| GAPIT | WSPCE1 | AX-93751738 | 8 | 8,016,517 | 4.91E-06 | 0.101 |
| GAPIT | WSPCE2 | AX-93751738 | 8 | 8,016,517 | 4.88E-06 | 0.090 |
| GAPIT | WSPCE5 | AX-93751738 | 8 | 8,016,517 | 1.10E-08 | 0.198 |
| GAPIT | WSPCE6 | AX-93751738 | 8 | 8,016,517 | 9.88E-10 | 0.237 |
| GAPIT | WSPCBLUP | AX-93751738 | 8 | 8,016,517 | 4.14E-07 | 0.304 |
| GAPIT | WSPCE5 | AX-94048021 | 8 | 8,028,255 | 6.31E-07 | 0.151 |
| GAPIT | WSPCE6 | AX-94048021 | 8 | 8,028,255 | 7.89E-08 | 0.183 |
| GAPIT | WSPCE5 | AX-93751745 | 8 | 8,037,117 | 8.36E-10 | 0.229 |
| GAPIT | WSPCE6 | AX-93751745 | 8 | 8,037,117 | 1.22E-10 | 0.264 |
| GAPIT | WSPCE5 | AX-93751760 | 8 | 8,071,376 | 1.80E-07 | 0.165 |
| GAPIT | WSPCE6 | AX-93751760 | 8 | 8,071,376 | 7.85E-08 | 0.183 |
| GAPIT | WSPCBLUP | AX-93751760 | 8 | 8,071,376 | 3.41E-06 | 0.336 |
| GAPIT | WSPCE5 | AX-93751767 | 8 | 8,087,995 | 6.38E-10 | 0.232 |
| GAPIT | WSPCE6 | AX-93751767 | 8 | 8,087,995 | 1.69E-11 | 0.290 |
| GAPIT | WSPCBLUP | AX-93751767 | 8 | 8,087,995 | 3.23E-06 | 0.267 |
| GAPIT | WSPCE5 | AX-93751782 | 8 | 8,127,066 | 7.22E-08 | 0.176 |
| GAPIT | WSPCE6 | AX-93751782 | 8 | 8,127,066 | 1.53E-08 | 0.203 |
| GAPIT | WSPCBLUP | AX-93751782 | 8 | 8,127,066 | 2.53E-06 | 0.333 |
| GAPIT | WSPCE5 | AX-93751793 | 8 | 8,157,793 | 7.65E-08 | 0.177 |
| GAPIT | WSPCE6 | AX-93751793 | 8 | 8,157,793 | 1.88E-08 | 0.202 |
| GAPIT | WSPCBLUP | AX-93751793 | 8 | 8,157,793 | 3.11E-06 | 0.328 |
| GAPIT | WSPCE5 | AX-94048075 | 8 | 8,166,922 | 4.03E-07 | 0.161 |
| GAPIT | WSPCE6 | AX-94048075 | 8 | 8,166,922 | 4.32E-08 | 0.193 |
| GAPIT | WSPCBLUP | AX-94048075 | 8 | 8,166,922 | 2.44E-06 | 0.326 |
| GAPIT | WSPCE1 | AX-94048076 | 8 | 8,184,086 | 1.93E-07 | 0.118 |
| GAPIT | WSPCE2 | AX-94048076 | 8 | 8,184,086 | 1.10E-07 | 0.123 |
| GAPIT | WSPCE3 | AX-94048076 | 8 | 8,184,086 | 9.59E-07 | 0.110 |
| GAPIT | WSPCE4 | AX-94048076 | 8 | 8,184,086 | 3.00E-06 | 0.102 |
| GAPIT | WSPCE5 | AX-94048076 | 8 | 8,184,086 | 5.46E-11 | 0.263 |
| GAPIT | WSPCE6 | AX-94048076 | 8 | 8,184,086 | 4.49E-12 | 0.308 |
| GAPIT | WSPCBLUP | AX-94048076 | 8 | 8,184,086 | 6.64E-10 | 0.277 |
| GAPIT | WSPCE1 | AX-94284415 | 8 | 8,185,799 | 1.40E-06 | 0.103 |
| GAPIT | WSPCE2 | AX-94284415 | 8 | 8,185,799 | 1.48E-06 | 0.104 |
| GAPIT | WSPCE6 | AX-94284415 | 8 | 8,185,799 | 1.62E-06 | 0.147 |
| GAPIT | WSPCBLUP | AX-94284415 | 8 | 8,185,799 | 3.89E-07 | 0.233 |
| GAPIT | WSPCE1 | AX-93634497 | 8 | 8,188,213 | 1.40E-06 | 0.103 |
| GAPIT | WSPCE2 | AX-93634497 | 8 | 8,188,213 | 1.48E-06 | 0.104 |
| GAPIT | WSPCE6 | AX-93634497 | 8 | 8,188,213 | 1.62E-06 | 0.147 |
| GAPIT | WSPCBLUP | AX-93634497 | 8 | 8,188,213 | 3.89E-07 | 0.233 |
| GAPIT | WSPCE1 | AX-93751799 | 8 | 8,191,120 | 2.71E-07 | 0.115 |
| GAPIT | WSPCE2 | AX-93751799 | 8 | 8,191,120 | 1.34E-07 | 0.122 |
| GAPIT | WSPCE3 | AX-93751799 | 8 | 8,191,120 | 1.02E-06 | 0.110 |
| GAPIT | WSPCE4 | AX-93751799 | 8 | 8,191,120 | 3.37E-06 | 0.101 |
| GAPIT | WSPCE5 | AX-93751799 | 8 | 8,191,120 | 4.38E-11 | 0.266 |
| GAPIT | WSPCE6 | AX-93751799 | 8 | 8,191,120 | 4.49E-12 | 0.308 |
| GAPIT | WSPCBLUP | AX-93751799 | 8 | 8,191,120 | 1.05E-09 | 0.279 |
| GAPIT | WSPCE1 | AX-94048080 | 8 | 8,192,241 | 1.71E-06 | 0.102 |
| GAPIT | WSPCE2 | AX-94048080 | 8 | 8,192,241 | 2.21E-06 | 0.102 |
| GAPIT | WSPCE6 | AX-94048080 | 8 | 8,192,241 | 7.07E-07 | 0.156 |
| GAPIT | WSPCBLUP | AX-94048080 | 8 | 8,192,241 | 4.53E-07 | 0.235 |
| GAPIT | WSPCE1 | AX-94048081 | 8 | 8,193,755 | 1.93E-07 | 0.118 |
| GAPIT | WSPCE2 | AX-94048081 | 8 | 8,193,755 | 1.10E-07 | 0.123 |
| GAPIT | WSPCE3 | AX-94048081 | 8 | 8,193,755 | 9.59E-07 | 0.110 |
| GAPIT | WSPCE4 | AX-94048081 | 8 | 8,193,755 | 3.00E-06 | 0.102 |
| GAPIT | WSPCE5 | AX-94048081 | 8 | 8,193,755 | 5.46E-11 | 0.263 |
| GAPIT | WSPCE6 | AX-94048081 | 8 | 8,193,755 | 4.49E-12 | 0.308 |
| GAPIT | WSPCBLUP | AX-94048081 | 8 | 8,193,755 | 6.64E-10 | 0.277 |
| GAPIT | WSPCE1 | AX-93634498 | 8 | 8,193,904 | 1.71E-06 | 0.102 |
| GAPIT | WSPCE2 | AX-93634498 | 8 | 8,193,904 | 2.21E-06 | 0.102 |
| GAPIT | WSPCE6 | AX-93634498 | 8 | 8,193,904 | 7.07E-07 | 0.156 |
| GAPIT | WSPCBLUP | AX-93634498 | 8 | 8,193,904 | 4.53E-07 | 0.235 |
| GAPIT | WSPCE1 | AX-94048082 | 8 | 8,195,739 | 1.71E-06 | 0.102 |
| GAPIT | WSPCE2 | AX-94048082 | 8 | 8,195,739 | 2.21E-06 | 0.102 |
| GAPIT | WSPCE6 | AX-94048082 | 8 | 8,195,739 | 7.07E-07 | 0.156 |
| GAPIT | WSPCBLUP | AX-94048082 | 8 | 8,195,739 | 4.53E-07 | 0.235 |
| GAPIT | WSPCE1 | AX-94048083 | 8 | 8,196,948 | 1.71E-06 | 0.102 |
| GAPIT | WSPCE2 | AX-94048083 | 8 | 8,196,948 | 2.21E-06 | 0.102 |
| GAPIT | WSPCE6 | AX-94048083 | 8 | 8,196,948 | 7.07E-07 | 0.156 |
| GAPIT | WSPCBLUP | AX-94048083 | 8 | 8,196,948 | 4.53E-07 | 0.235 |
| GAPIT | WSPCE1 | AX-94048084 | 8 | 8,198,583 | 1.71E-06 | 0.102 |
| GAPIT | WSPCE2 | AX-94048084 | 8 | 8,198,583 | 2.21E-06 | 0.102 |
| GAPIT | WSPCE6 | AX-94048084 | 8 | 8,198,583 | 7.07E-07 | 0.156 |
| GAPIT | WSPCBLUP | AX-94048084 | 8 | 8,198,583 | 4.53E-07 | 0.235 |
| GAPIT | WSPCE1 | AX-93751805 | 8 | 8,200,391 | 1.78E-06 | 0.102 |
| GAPIT | WSPCE2 | AX-93751805 | 8 | 8,200,391 | 1.88E-06 | 0.103 |
| GAPIT | WSPCE3 | AX-93751805 | 8 | 8,200,391 | 4.11E-06 | 0.100 |
| GAPIT | WSPCE6 | AX-93751805 | 8 | 8,200,391 | 3.05E-07 | 0.168 |
| GAPIT | WSPCBLUP | AX-93751805 | 8 | 8,200,391 | 4.53E-07 | 0.235 |
| GAPIT | WSPCE1 | AX-93751806 | 8 | 8,201,818 | 3.34E-07 | 0.100 |
| GAPIT | WSPCE2 | AX-93751806 | 8 | 8,201,818 | 3.56E-07 | 0.101 |
| GAPIT | WSPCE3 | AX-93751806 | 8 | 8,201,818 | 1.13E-06 | 0.095 |
| GAPIT | WSPCE4 | AX-93751806 | 8 | 8,201,818 | 1.15E-06 | 0.095 |
| GAPIT | WSPCE6 | AX-93751806 | 8 | 8,201,818 | 4.46E-08 | 0.168 |
| GAPIT | WSPCBLUP | AX-93751806 | 8 | 8,201,818 | 2.77E-07 | 0.238 |
| GAPIT | WSPCE1 | AX-94048086 | 8 | 8,204,675 | 3.70E-08 | 0.115 |
| GAPIT | WSPCE2 | AX-94048086 | 8 | 8,204,675 | 1.78E-08 | 0.122 |
| GAPIT | WSPCE3 | AX-94048086 | 8 | 8,204,675 | 1.45E-07 | 0.110 |
| GAPIT | WSPCE4 | AX-94048086 | 8 | 8,204,675 | 5.07E-07 | 0.101 |
| GAPIT | WSPCE5 | AX-94048086 | 8 | 8,204,675 | 7.75E-12 | 0.261 |
| GAPIT | WSPCE6 | AX-94048086 | 8 | 8,204,675 | 8.46E-13 | 0.302 |
| GAPIT | WSPCBLUP | AX-94048086 | 8 | 8,204,675 | 4.72E-10 | 0.275 |
| GAPIT | WSPCE1 | AX-94048089 | 8 | 8,212,342 | 3.18E-06 | 0.098 |
| GAPIT | WSPCE2 | AX-94048089 | 8 | 8,212,342 | 2.12E-06 | 0.102 |
| GAPIT | WSPCE5 | AX-94048089 | 8 | 8,212,342 | 6.69E-10 | 0.232 |
| GAPIT | WSPCE6 | AX-94048089 | 8 | 8,212,342 | 6.80E-11 | 0.272 |
| GAPIT | WSPCBLUP | AX-94048089 | 8 | 8,212,342 | 1.27E-08 | 0.272 |
| GAPIT | WSPCE5 | AX-93751813 | 8 | 8,216,777 | 1.60E-07 | 0.176 |
| GAPIT | WSPCE6 | AX-93751813 | 8 | 8,216,777 | 4.27E-09 | 0.237 |
| GAPIT | WSPCBLUP | AX-93751813 | 8 | 8,216,777 | 4.32E-07 | 0.255 |
| GAPIT | WSPCE1 | AX-94048095 | 8 | 8,224,100 | 2.51E-07 | 0.116 |
| GAPIT | WSPCE2 | AX-94048095 | 8 | 8,224,100 | 1.33E-07 | 0.122 |
| GAPIT | WSPCE3 | AX-94048095 | 8 | 8,224,100 | 9.65E-07 | 0.110 |
| GAPIT | WSPCE4 | AX-94048095 | 8 | 8,224,100 | 2.82E-06 | 0.102 |
| GAPIT | WSPCE5 | AX-94048095 | 8 | 8,224,100 | 5.54E-11 | 0.263 |
| GAPIT | WSPCE6 | AX-94048095 | 8 | 8,224,100 | 6.58E-12 | 0.303 |
| GAPIT | WSPCBLUP | AX-94048095 | 8 | 8,224,100 | 9.15E-10 | 0.279 |
| GAPIT | WSPCE1 | AX-93751817 | 8 | 8,225,628 | 4.71E-07 | 0.110 |
| GAPIT | WSPCE2 | AX-93751817 | 8 | 8,225,628 | 4.45E-07 | 0.115 |
| GAPIT | WSPCE3 | AX-93751817 | 8 | 8,225,628 | 3.86E-06 | 0.101 |
| GAPIT | WSPCE5 | AX-93751817 | 8 | 8,225,628 | 4.45E-10 | 0.238 |
| GAPIT | WSPCE6 | AX-93751817 | 8 | 8,225,628 | 4.21E-11 | 0.282 |
| GAPIT | WSPCBLUP | AX-93751817 | 8 | 8,225,628 | 4.70E-09 | 0.270 |
| GAPIT | WSPCE1 | AX-93930665 | 8 | 8,227,016 | 2.06E-07 | 0.118 |
| GAPIT | WSPCE2 | AX-93930665 | 8 | 8,227,016 | 1.18E-07 | 0.123 |
| GAPIT | WSPCE3 | AX-93930665 | 8 | 8,227,016 | 9.98E-07 | 0.110 |
| GAPIT | WSPCE4 | AX-93930665 | 8 | 8,227,016 | 3.15E-06 | 0.102 |
| GAPIT | WSPCE5 | AX-93930665 | 8 | 8,227,016 | 5.48E-11 | 0.263 |
| GAPIT | WSPCE6 | AX-93930665 | 8 | 8,227,016 | 4.54E-12 | 0.308 |
| GAPIT | WSPCBLUP | AX-93930665 | 8 | 8,227,016 | 5.57E-10 | 0.279 |
| GAPIT | WSPCE2 | AX-93751818 | 8 | 8,228,669 | 1.04E-06 | 0.107 |
| GAPIT | WSPCE3 | AX-93751818 | 8 | 8,228,669 | 3.73E-06 | 0.100 |
| GAPIT | WSPCE6 | AX-93751818 | 8 | 8,228,669 | 1.38E-06 | 0.157 |
| GAPIT | WSPCBLUP | AX-93751818 | 8 | 8,228,669 | 3.89E-07 | 0.233 |
| GAPIT | WSPCE1 | AX-94048102 | 8 | 8,236,728 | 1.93E-07 | 0.118 |
| GAPIT | WSPCE2 | AX-94048102 | 8 | 8,236,728 | 1.10E-07 | 0.123 |
| GAPIT | WSPCE3 | AX-94048102 | 8 | 8,236,728 | 9.59E-07 | 0.110 |
| GAPIT | WSPCE4 | AX-94048102 | 8 | 8,236,728 | 3.00E-06 | 0.102 |
| GAPIT | WSPCE5 | AX-94048102 | 8 | 8,236,728 | 5.46E-11 | 0.263 |
| GAPIT | WSPCE6 | AX-94048102 | 8 | 8,236,728 | 4.49E-12 | 0.308 |
| GAPIT | WSPCBLUP | AX-94048102 | 8 | 8,236,728 | 6.64E-10 | 0.277 |
| GAPIT | WSPCE1 | AX-93634502 | 8 | 8,240,007 | 1.93E-07 | 0.118 |
| GAPIT | WSPCE2 | AX-93634502 | 8 | 8,240,007 | 1.10E-07 | 0.123 |
| GAPIT | WSPCE3 | AX-93634502 | 8 | 8,240,007 | 9.59E-07 | 0.110 |
| GAPIT | WSPCE4 | AX-93634502 | 8 | 8,240,007 | 3.00E-06 | 0.102 |
| GAPIT | WSPCE5 | AX-93634502 | 8 | 8,240,007 | 5.46E-11 | 0.263 |
| GAPIT | WSPCE6 | AX-93634502 | 8 | 8,240,007 | 4.49E-12 | 0.308 |
| GAPIT | WSPCBLUP | AX-93634502 | 8 | 8,240,007 | 6.64E-10 | 0.277 |
| GAPIT | WSPCE1 | AX-93751824 | 8 | 8,241,205 | 7.08E-07 | 0.109 |
| GAPIT | WSPCE2 | AX-93751824 | 8 | 8,241,205 | 4.52E-07 | 0.114 |
| GAPIT | WSPCE3 | AX-93751824 | 8 | 8,241,205 | 4.26E-06 | 0.100 |
| GAPIT | WSPCE5 | AX-93751824 | 8 | 8,241,205 | 3.63E-10 | 0.246 |
| GAPIT | WSPCE6 | AX-93751824 | 8 | 8,241,205 | 1.40E-11 | 0.298 |
| GAPIT | WSPCBLUP | AX-93751824 | 8 | 8,241,205 | 2.86E-09 | 0.272 |
| GAPIT | WSPCE5 | AX-94048104 | 8 | 8,247,379 | 4.03E-07 | 0.161 |
| GAPIT | WSPCE6 | AX-94048104 | 8 | 8,247,379 | 4.32E-08 | 0.193 |
| GAPIT | WSPCBLUP | AX-94048104 | 8 | 8,247,379 | 2.44E-06 | 0.326 |
| GAPIT | WSPCE5 | AX-94048105 | 8 | 8,251,019 | 7.07E-07 | 0.150 |
| GAPIT | WSPCE6 | AX-94048105 | 8 | 8,251,019 | 1.34E-07 | 0.176 |
| GAPIT | WSPCBLUP | AX-94048105 | 8 | 8,251,019 | 2.44E-06 | 0.326 |
| GAPIT | WSPCE1 | AX-93751829 | 8 | 8,255,498 | 1.93E-07 | 0.118 |
| GAPIT | WSPCE2 | AX-93751829 | 8 | 8,255,498 | 1.10E-07 | 0.123 |
| GAPIT | WSPCE3 | AX-93751829 | 8 | 8,255,498 | 9.59E-07 | 0.110 |
| GAPIT | WSPCE4 | AX-93751829 | 8 | 8,255,498 | 3.00E-06 | 0.102 |
| GAPIT | WSPCE5 | AX-93751829 | 8 | 8,255,498 | 5.46E-11 | 0.263 |
| GAPIT | WSPCE6 | AX-93751829 | 8 | 8,255,498 | 4.49E-12 | 0.308 |
| GAPIT | WSPCBLUP | AX-93751829 | 8 | 8,255,498 | 6.64E-10 | 0.277 |
| GAPIT | WSPCE1 | AX-93751831 | 8 | 8,260,590 | 1.93E-07 | 0.118 |
| GAPIT | WSPCE2 | AX-93751831 | 8 | 8,260,590 | 1.10E-07 | 0.123 |
| GAPIT | WSPCE3 | AX-93751831 | 8 | 8,260,590 | 9.59E-07 | 0.110 |
| GAPIT | WSPCE4 | AX-93751831 | 8 | 8,260,590 | 3.00E-06 | 0.102 |
| GAPIT | WSPCE5 | AX-93751831 | 8 | 8,260,590 | 5.46E-11 | 0.263 |
| GAPIT | WSPCE6 | AX-93751831 | 8 | 8,260,590 | 4.49E-12 | 0.308 |
| GAPIT | WSPCBLUP | AX-93751831 | 8 | 8,260,590 | 6.64E-10 | 0.277 |
| GAPIT | WSPCE5 | AX-93634504 | 8 | 8,268,861 | 9.40E-07 | 0.147 |
| GAPIT | WSPCE6 | AX-93634504 | 8 | 8,268,861 | 3.98E-07 | 0.165 |
| GAPIT | WSPCBLUP | AX-93634504 | 8 | 8,268,861 | 4.67E-06 | 0.338 |
| GAPIT | WSPCE5 | AX-93930669 | 8 | 8,272,057 | 1.14E-11 | 0.257 |
| GAPIT | WSPCE6 | AX-93930669 | 8 | 8,272,057 | 9.62E-14 | 0.333 |
| GAPIT | WSPCBLUP | AX-93930669 | 8 | 8,272,057 | 9.42E-08 | 0.248 |
| GAPIT | WSPCE5 | AX-94048117 | 8 | 8,275,292 | 1.14E-11 | 0.257 |
| GAPIT | WSPCE6 | AX-94048117 | 8 | 8,275,292 | 9.62E-14 | 0.333 |
| GAPIT | WSPCBLUP | AX-94048117 | 8 | 8,275,292 | 9.42E-08 | 0.248 |
| GAPIT | WSPCE5 | AX-93634506 | 8 | 8,281,564 | 7.80E-11 | 0.259 |
| GAPIT | WSPCE6 | AX-93634506 | 8 | 8,281,564 | 5.19E-13 | 0.338 |
| GAPIT | WSPCBLUP | AX-93634506 | 8 | 8,281,564 | 9.42E-08 | 0.248 |
| GAPIT | WSPCE5 | AX-93751849 | 8 | 8,301,839 | 5.11E-11 | 0.264 |
| GAPIT | WSPCE6 | AX-93751849 | 8 | 8,301,839 | 8.79E-13 | 0.330 |
| GAPIT | WSPCBLUP | AX-93751849 | 8 | 8,301,839 | 6.14E-07 | 0.248 |
| GAPIT | WSPCE5 | AX-93751851 | 8 | 8,305,731 | 5.11E-11 | 0.264 |
| GAPIT | WSPCE6 | AX-93751851 | 8 | 8,305,731 | 8.79E-13 | 0.330 |
| GAPIT | WSPCBLUP | AX-93751851 | 8 | 8,305,731 | 6.14E-07 | 0.248 |
| GAPIT | WSPCE5 | AX-93751852 | 8 | 8,307,665 | 6.72E-11 | 0.262 |
| GAPIT | WSPCE6 | AX-93751852 | 8 | 8,307,665 | 1.43E-12 | 0.328 |
| GAPIT | WSPCBLUP | AX-93751852 | 8 | 8,307,665 | 1.37E-06 | 0.250 |
| GAPIT | WSPCE5 | AX-93751853 | 8 | 8,308,891 | 5.11E-11 | 0.264 |
| GAPIT | WSPCE6 | AX-93751853 | 8 | 8,308,891 | 8.79E-13 | 0.330 |
| GAPIT | WSPCBLUP | AX-93751853 | 8 | 8,308,891 | 6.14E-07 | 0.248 |
| GAPIT | WSPCE5 | AX-93751854 | 8 | 8,310,464 | 7.41E-12 | 0.262 |
| GAPIT | WSPCE6 | AX-93751854 | 8 | 8,310,464 | 1.65E-13 | 0.326 |
| GAPIT | WSPCBLUP | AX-93751854 | 8 | 8,310,464 | 6.14E-07 | 0.248 |
| GAPIT | WSPCE5 | AX-93751856 | 8 | 8,317,852 | 6.72E-11 | 0.262 |
| GAPIT | WSPCE6 | AX-93751856 | 8 | 8,317,852 | 1.43E-12 | 0.328 |
| GAPIT | WSPCBLUP | AX-93751856 | 8 | 8,317,852 | 1.37E-06 | 0.250 |
| GAPIT | WSPCE5 | AX-93751857 | 8 | 8,320,068 | 4.41E-11 | 0.266 |
| GAPIT | WSPCE6 | AX-93751857 | 8 | 8,320,068 | 7.42E-13 | 0.334 |
| GAPIT | WSPCBLUP | AX-93751857 | 8 | 8,320,068 | 7.55E-07 | 0.245 |
| GAPIT | WSPCE5 | AX-94048138 | 8 | 8,321,649 | 1.52E-10 | 0.248 |
| GAPIT | WSPCE6 | AX-94048138 | 8 | 8,321,649 | 4.89E-12 | 0.326 |
| GAPIT | WSPCBLUP | AX-94048138 | 8 | 8,321,649 | 6.49E-07 | 0.238 |
| GAPIT | WSPCE1 | AX-94048146 | 8 | 8,356,185 | 4.10E-06 | 0.096 |
| GAPIT | WSPCE2 | AX-94048146 | 8 | 8,356,185 | 1.05E-06 | 0.107 |
| GAPIT | WSPCE3 | AX-94048146 | 8 | 8,356,185 | 4.64E-06 | 0.098 |
| GAPIT | WSPCE4 | AX-94048146 | 8 | 8,356,185 | 2.02E-06 | 0.105 |
| GAPIT | WSPCE1 | AX-94048148 | 8 | 8,368,519 | 3.91E-08 | 0.115 |
| GAPIT | WSPCE2 | AX-94048148 | 8 | 8,368,519 | 2.15E-08 | 0.120 |
| GAPIT | WSPCE3 | AX-94048148 | 8 | 8,368,519 | 4.97E-07 | 0.101 |
| GAPIT | WSPCE5 | AX-94048148 | 8 | 8,368,519 | 2.86E-11 | 0.245 |
| GAPIT | WSPCE6 | AX-94048148 | 8 | 8,368,519 | 5.90E-13 | 0.306 |
| GAPIT | WSPCBLUP | AX-94048148 | 8 | 8,368,519 | 1.82E-08 | 0.265 |
| GAPIT | WSPCE1 | AX-94048149 | 8 | 8,372,206 | 1.33E-07 | 0.106 |
| GAPIT | WSPCE2 | AX-94048149 | 8 | 8,372,206 | 7.41E-08 | 0.112 |
| GAPIT | WSPCE3 | AX-94048149 | 8 | 8,372,206 | 1.73E-06 | 0.092 |
| GAPIT | WSPCE5 | AX-94048149 | 8 | 8,372,206 | 1.02E-10 | 0.235 |
| GAPIT | WSPCE6 | AX-94048149 | 8 | 8,372,206 | 1.69E-12 | 0.297 |
| GAPIT | WSPCBLUP | AX-94048149 | 8 | 8,372,206 | 4.23E-08 | 0.262 |
| GAPIT | WSPCE1 | AX-94048150 | 8 | 8,373,459 | 7.84E-07 | 0.094 |
| GAPIT | WSPCE2 | AX-94048150 | 8 | 8,373,459 | 8.41E-07 | 0.095 |
| GAPIT | WSPCE5 | AX-94048150 | 8 | 8,373,459 | 3.21E-10 | 0.216 |
| GAPIT | WSPCE6 | AX-94048150 | 8 | 8,373,459 | 5.64E-11 | 0.247 |
| GAPIT | WSPCBLUP | AX-94048150 | 8 | 8,373,459 | 3.17E-07 | 0.225 |
| GAPIT | WSPCE1 | AX-94048153 | 8 | 8,376,734 | 2.00E-06 | 0.088 |
| GAPIT | WSPCE2 | AX-94048153 | 8 | 8,376,734 | 1.74E-06 | 0.090 |
| GAPIT | WSPCE5 | AX-94048153 | 8 | 8,376,734 | 1.03E-10 | 0.230 |
| GAPIT | WSPCE6 | AX-94048153 | 8 | 8,376,734 | 2.22E-11 | 0.261 |
| GAPIT | WSPCBLUP | AX-94048153 | 8 | 8,376,734 | 5.17E-08 | 0.223 |
| GAPIT | WSPCE1 | AX-93751870 | 8 | 8,378,286 | 4.26E-07 | 0.100 |
| GAPIT | WSPCE2 | AX-93751870 | 8 | 8,378,286 | 3.73E-07 | 0.102 |
| GAPIT | WSPCE5 | AX-93751870 | 8 | 8,378,286 | 4.53E-10 | 0.216 |
| GAPIT | WSPCE6 | AX-93751870 | 8 | 8,378,286 | 1.30E-12 | 0.300 |
| GAPIT | WSPCBLUP | AX-93751870 | 8 | 8,378,286 | 2.97E-07 | 0.243 |
| GAPIT | WSPCE1 | AX-93634511 | 8 | 8,378,380 | 3.66E-06 | 0.101 |
| GAPIT | WSPCE2 | AX-93634511 | 8 | 8,378,380 | 3.50E-06 | 0.102 |
| GAPIT | WSPCE5 | AX-93634511 | 8 | 8,378,380 | 5.64E-11 | 0.276 |
| GAPIT | WSPCE6 | AX-93634511 | 8 | 8,378,380 | 3.63E-11 | 0.286 |
| GAPIT | WSPCBLUP | AX-93634511 | 8 | 8,378,380 | 4.51E-07 | 0.213 |
| GAPIT | WSPCE1 | AX-94048155 | 8 | 8,386,661 | 3.58E-08 | 0.115 |
| GAPIT | WSPCE2 | AX-94048155 | 8 | 8,386,661 | 3.48E-08 | 0.117 |
| GAPIT | WSPCE3 | AX-94048155 | 8 | 8,386,661 | 2.68E-07 | 0.105 |
| GAPIT | WSPCE5 | AX-94048155 | 8 | 8,386,661 | 1.95E-11 | 0.250 |
| GAPIT | WSPCE6 | AX-94048155 | 8 | 8,386,661 | 2.82E-12 | 0.286 |
| GAPIT | WSPCBLUP | AX-94048155 | 8 | 8,386,661 | 1.46E-08 | 0.230 |
| GAPIT | WSPCE5 | AX-94048156 | 8 | 8,388,580 | 8.46E-10 | 0.205 |
| GAPIT | WSPCE6 | AX-94048156 | 8 | 8,388,580 | 4.17E-10 | 0.223 |
| GAPIT | WSPCE1 | AX-93930676 | 8 | 8,396,392 | 3.91E-08 | 0.115 |
| GAPIT | WSPCE2 | AX-93930676 | 8 | 8,396,392 | 2.15E-08 | 0.120 |
| GAPIT | WSPCE3 | AX-93930676 | 8 | 8,396,392 | 4.97E-07 | 0.101 |
| GAPIT | WSPCE5 | AX-93930676 | 8 | 8,396,392 | 2.86E-11 | 0.245 |
| GAPIT | WSPCE6 | AX-93930676 | 8 | 8,396,392 | 5.90E-13 | 0.306 |
| GAPIT | WSPCBLUP | AX-93930676 | 8 | 8,396,392 | 1.82E-08 | 0.265 |
| GAPIT | WSPCE1 | AX-94048158 | 8 | 8,408,044 | 9.38E-07 | 0.093 |
| GAPIT | WSPCE2 | AX-94048158 | 8 | 8,408,044 | 6.22E-07 | 0.097 |
| GAPIT | WSPCE5 | AX-94048158 | 8 | 8,408,044 | 1.94E-10 | 0.222 |
| GAPIT | WSPCE6 | AX-94048158 | 8 | 8,408,044 | 1.15E-11 | 0.268 |
| GAPIT | WSPCBLUP | AX-94048158 | 8 | 8,408,044 | 8.84E-08 | 0.250 |
| GAPIT | WSPCE1 | AX-93751880 | 8 | 8,417,640 | 5.27E-08 | 0.115 |
| GAPIT | WSPCE2 | AX-93751880 | 8 | 8,417,640 | 2.93E-08 | 0.121 |
| GAPIT | WSPCE3 | AX-93751880 | 8 | 8,417,640 | 6.49E-07 | 0.101 |
| GAPIT | WSPCE5 | AX-93751880 | 8 | 8,417,640 | 2.86E-11 | 0.245 |
| GAPIT | WSPCE6 | AX-93751880 | 8 | 8,417,640 | 6.66E-13 | 0.305 |
| GAPIT | WSPCBLUP | AX-93751880 | 8 | 8,417,640 | 3.28E-08 | 0.262 |
| GAPIT | WSPCE1 | AX-93751882 | 8 | 8,422,602 | 1.58E-08 | 0.120 |
| GAPIT | WSPCE2 | AX-93751882 | 8 | 8,422,602 | 1.27E-08 | 0.124 |
| GAPIT | WSPCE3 | AX-93751882 | 8 | 8,422,602 | 9.62E-08 | 0.112 |
| GAPIT | WSPCE5 | AX-93751882 | 8 | 8,422,602 | 1.80E-11 | 0.250 |
| GAPIT | WSPCE6 | AX-93751882 | 8 | 8,422,602 | 2.27E-12 | 0.294 |
| GAPIT | WSPCBLUP | AX-93751882 | 8 | 8,422,602 | 3.87E-08 | 0.225 |
| GAPIT | WSPCE1 | AX-93751883 | 8 | 8,425,568 | 2.72E-08 | 0.120 |
| GAPIT | WSPCE2 | AX-93751883 | 8 | 8,425,568 | 2.09E-08 | 0.124 |
| GAPIT | WSPCE3 | AX-93751883 | 8 | 8,425,568 | 1.43E-07 | 0.113 |
| GAPIT | WSPCE5 | AX-93751883 | 8 | 8,425,568 | 1.85E-11 | 0.254 |
| GAPIT | WSPCE6 | AX-93751883 | 8 | 8,425,568 | 6.10E-12 | 0.280 |
| GAPIT | WSPCBLUP | AX-93751883 | 8 | 8,425,568 | 1.63E-08 | 0.230 |
| GAPIT | WSPCE1 | AX-94284412 | 8 | 8,427,110 | 1.89E-08 | 0.120 |
| GAPIT | WSPCE2 | AX-94284412 | 8 | 8,427,110 | 1.47E-08 | 0.124 |
| GAPIT | WSPCE3 | AX-94284412 | 8 | 8,427,110 | 1.18E-07 | 0.112 |
| GAPIT | WSPCE5 | AX-94284412 | 8 | 8,427,110 | 7.07E-14 | 0.321 |
| GAPIT | WSPCE6 | AX-94284412 | 8 | 8,427,110 | 7.21E-14 | 0.335 |
| GAPIT | WSPCBLUP | AX-94284412 | 8 | 8,427,110 | 4.29E-09 | 0.223 |
| GAPIT | WSPCE1 | AX-94048171 | 8 | 8,439,795 | 1.45E-09 | 0.137 |
| GAPIT | WSPCE2 | AX-94048171 | 8 | 8,439,795 | 1.41E-09 | 0.139 |
| GAPIT | WSPCE3 | AX-94048171 | 8 | 8,439,795 | 1.50E-08 | 0.126 |
| GAPIT | WSPCE4 | AX-94048171 | 8 | 8,439,795 | 8.25E-07 | 0.097 |
| GAPIT | WSPCE5 | AX-94048171 | 8 | 8,439,795 | 9.00E-13 | 0.288 |
| GAPIT | WSPCE6 | AX-94048171 | 8 | 8,439,795 | 1.51E-12 | 0.294 |
| GAPIT | WSPCBLUP | AX-94048171 | 8 | 8,439,795 | 5.95E-10 | 0.235 |
| GAPIT | WSPCE5 | AX-93751888 | 8 | 8,440,896 | 3.63E-12 | 0.270 |
| GAPIT | WSPCE6 | AX-93751888 | 8 | 8,440,896 | 4.42E-11 | 0.250 |
| GAPIT | WSPCBLUP | AX-93751888 | 8 | 8,440,896 | 9.95E-07 | 0.211 |
| GAPIT | WSPCE1 | AX-94048173 | 8 | 8,442,251 | 5.09E-07 | 0.097 |
| GAPIT | WSPCE2 | AX-94048173 | 8 | 8,442,251 | 4.39E-07 | 0.099 |
| GAPIT | WSPCE3 | AX-94048173 | 8 | 8,442,251 | 2.61E-06 | 0.089 |
| GAPIT | WSPCE5 | AX-94048173 | 8 | 8,442,251 | 1.92E-10 | 0.222 |
| GAPIT | WSPCE6 | AX-94048173 | 8 | 8,442,251 | 6.68E-11 | 0.245 |
| GAPIT | WSPCBLUP | AX-94048173 | 8 | 8,442,251 | 2.27E-07 | 0.221 |
| GAPIT | WSPCE1 | AX-93751891 | 8 | 8,445,259 | 3.74E-10 | 0.153 |
| GAPIT | WSPCE2 | AX-93751891 | 8 | 8,445,259 | 5.30E-10 | 0.153 |
| GAPIT | WSPCE3 | AX-93751891 | 8 | 8,445,259 | 4.62E-09 | 0.140 |
| GAPIT | WSPCE4 | AX-93751891 | 8 | 8,445,259 | 1.26E-07 | 0.117 |
| GAPIT | WSPCE5 | AX-93751891 | 8 | 8,445,259 | 9.67E-12 | 0.278 |
| GAPIT | WSPCE6 | AX-93751891 | 8 | 8,445,259 | 1.11E-11 | 0.284 |
| GAPIT | WSPCBLUP | AX-93751891 | 8 | 8,445,259 | 1.25E-08 | 0.250 |
| GAPIT | WSPCE1 | AX-94048176 | 8 | 8,446,615 | 2.97E-08 | 0.124 |
| GAPIT | WSPCE2 | AX-94048176 | 8 | 8,446,615 | 4.86E-08 | 0.121 |
| GAPIT | WSPCE3 | AX-94048176 | 8 | 8,446,615 | 2.66E-07 | 0.111 |
| GAPIT | WSPCE4 | AX-94048176 | 8 | 8,446,615 | 4.82E-06 | 0.089 |
| GAPIT | WSPCE5 | AX-94048176 | 8 | 8,446,615 | 3.48E-11 | 0.249 |
| GAPIT | WSPCE6 | AX-94048176 | 8 | 8,446,615 | 1.98E-11 | 0.275 |
| GAPIT | WSPCBLUP | AX-94048176 | 8 | 8,446,615 | 1.38E-07 | 0.218 |
| GAPIT | WSPCE1 | AX-94284416 | 8 | 8,462,762 | 1.64E-09 | 0.137 |
| GAPIT | WSPCE2 | AX-94284416 | 8 | 8,462,762 | 1.59E-09 | 0.139 |
| GAPIT | WSPCE3 | AX-94284416 | 8 | 8,462,762 | 1.65E-08 | 0.126 |
| GAPIT | WSPCE4 | AX-94284416 | 8 | 8,462,762 | 8.98E-07 | 0.097 |
| GAPIT | WSPCE5 | AX-94284416 | 8 | 8,462,762 | 1.23E-12 | 0.286 |
| GAPIT | WSPCE6 | AX-94284416 | 8 | 8,462,762 | 2.65E-12 | 0.291 |
| GAPIT | WSPCBLUP | AX-94284416 | 8 | 8,462,762 | 1.56E-09 | 0.238 |
| GAPIT | WSPCE1 | AX-94048179 | 8 | 8,464,344 | 1.45E-09 | 0.137 |
| GAPIT | WSPCE2 | AX-94048179 | 8 | 8,464,344 | 1.41E-09 | 0.139 |
| GAPIT | WSPCE3 | AX-94048179 | 8 | 8,464,344 | 1.50E-08 | 0.126 |
| GAPIT | WSPCE4 | AX-94048179 | 8 | 8,464,344 | 8.25E-07 | 0.097 |
| GAPIT | WSPCE5 | AX-94048179 | 8 | 8,464,344 | 9.00E-13 | 0.288 |
| GAPIT | WSPCE6 | AX-94048179 | 8 | 8,464,344 | 1.51E-12 | 0.294 |
| GAPIT | WSPCBLUP | AX-94048179 | 8 | 8,464,344 | 5.95E-10 | 0.235 |
| GAPIT | WSPCE1 | AX-93751896 | 8 | 8,466,035 | 3.91E-08 | 0.115 |
| GAPIT | WSPCE2 | AX-93751896 | 8 | 8,466,035 | 2.15E-08 | 0.120 |
| GAPIT | WSPCE3 | AX-93751896 | 8 | 8,466,035 | 4.97E-07 | 0.101 |
| GAPIT | WSPCE5 | AX-93751896 | 8 | 8,466,035 | 2.86E-11 | 0.245 |
| GAPIT | WSPCE6 | AX-93751896 | 8 | 8,466,035 | 5.90E-13 | 0.306 |
| GAPIT | WSPCBLUP | AX-93751896 | 8 | 8,466,035 | 1.82E-08 | 0.265 |
| GAPIT | WSPCE1 | AX-93662764 | 8 | 8,469,985 | 2.13E-08 | 0.119 |
| GAPIT | WSPCE2 | AX-93662764 | 8 | 8,469,985 | 1.61E-08 | 0.122 |
| GAPIT | WSPCE3 | AX-93662764 | 8 | 8,469,985 | 1.18E-07 | 0.111 |
| GAPIT | WSPCE5 | AX-93662764 | 8 | 8,469,985 | 1.12E-11 | 0.256 |
| GAPIT | WSPCE6 | AX-93662764 | 8 | 8,469,985 | 3.33E-12 | 0.284 |
| GAPIT | WSPCBLUP | AX-93662764 | 8 | 8,469,985 | 1.00E-08 | 0.225 |
| GAPIT | WSPCE5 | AX-93930678 | 8 | 8,483,831 | 1.49E-10 | 0.250 |
| GAPIT | WSPCE6 | AX-93930678 | 8 | 8,483,831 | 5.63E-13 | 0.331 |
| GAPIT | WSPCBLUP | AX-93930678 | 8 | 8,483,831 | 1.16E-06 | 0.255 |
| GAPIT | WSPCE5 | AX-93960561 | 8 | 8,484,848 | 5.15E-10 | 0.241 |
| GAPIT | WSPCE6 | AX-93960561 | 8 | 8,484,848 | 4.27E-11 | 0.278 |
| GAPIT | WSPCBLUP | AX-93960561 | 8 | 8,484,848 | 4.52E-06 | 0.260 |
| GAPIT | WSPCE5 | AX-93751901 | 8 | 8,500,850 | 2.01E-09 | 0.194 |
| GAPIT | WSPCE6 | AX-93751901 | 8 | 8,500,850 | 1.19E-11 | 0.267 |
| GAPIT | WSPCBLUP | AX-93751901 | 8 | 8,500,850 | 1.29E-06 | 0.275 |
| GAPIT | WSPCE5 | AX-94048185 | 8 | 8,514,566 | 1.49E-08 | 0.194 |
| GAPIT | WSPCE6 | AX-94048185 | 8 | 8,514,566 | 9.85E-11 | 0.267 |
| GAPIT | WSPCBLUP | AX-94048185 | 8 | 8,514,566 | 8.16E-07 | 0.277 |
| GAPIT | WSPCE5 | AX-93751903 | 8 | 8,532,575 | 2.01E-09 | 0.194 |
| GAPIT | WSPCE6 | AX-93751903 | 8 | 8,532,575 | 1.19E-11 | 0.267 |
| GAPIT | WSPCBLUP | AX-93751903 | 8 | 8,532,575 | 1.29E-06 | 0.275 |
| GAPIT | WSPCE5 | AX-93751904 | 8 | 8,534,949 | 2.01E-09 | 0.194 |
| GAPIT | WSPCE6 | AX-93751904 | 8 | 8,534,949 | 1.19E-11 | 0.267 |
| GAPIT | WSPCBLUP | AX-93751904 | 8 | 8,534,949 | 1.29E-06 | 0.275 |
| GAPIT | WSPCE1 | AX-93634527 | 8 | 8,577,294 | 9.06E-07 | 0.093 |
| GAPIT | WSPCE2 | AX-93634527 | 8 | 8,577,294 | 4.63E-07 | 0.099 |
| GAPIT | WSPCE3 | AX-93634527 | 8 | 8,577,294 | 3.21E-06 | 0.087 |
| GAPIT | WSPCE5 | AX-93634527 | 8 | 8,577,294 | 1.79E-10 | 0.223 |
| GAPIT | WSPCE6 | AX-93634527 | 8 | 8,577,294 | 8.07E-12 | 0.272 |
| GAPIT | WSPCBLUP | AX-93634527 | 8 | 8,577,294 | 7.05E-08 | 0.282 |
| GAPIT | WSPCE1 | AX-93751914 | 8 | 8,592,601 | 7.90E-10 | 0.141 |
| GAPIT | WSPCE2 | AX-93751914 | 8 | 8,592,601 | 5.83E-10 | 0.145 |
| GAPIT | WSPCE3 | AX-93751914 | 8 | 8,592,601 | 5.93E-09 | 0.132 |
| GAPIT | WSPCE4 | AX-93751914 | 8 | 8,592,601 | 4.21E-08 | 0.102 |
| GAPIT | WSPCE5 | AX-93751914 | 8 | 8,592,601 | 5.56E-14 | 0.294 |
| GAPIT | WSPCE6 | AX-93751914 | 8 | 8,592,601 | 1.95E-12 | 0.291 |
| GAPIT | WSPCBLUP | AX-93751914 | 8 | 8,592,601 | 4.30E-13 | 0.230 |
| GAPIT | WSPCE1 | AX-94048203 | 8 | 8,626,441 | 4.07E-06 | 0.096 |
| GAPIT | WSPCE2 | AX-94048203 | 8 | 8,626,441 | 3.21E-06 | 0.099 |
| GAPIT | WSPCE5 | AX-94048203 | 8 | 8,626,441 | 7.23E-10 | 0.231 |
| GAPIT | WSPCE6 | AX-94048203 | 8 | 8,626,441 | 2.87E-09 | 0.224 |
| GAPIT | WSPCBLUP | AX-94048203 | 8 | 8,626,441 | 3.00E-07 | 0.243 |
| GAPIT | WSPCE5 | AX-94284428 | 8 | 8,627,848 | 2.11E-11 | 0.278 |
| GAPIT | WSPCE6 | AX-94284428 | 8 | 8,627,848 | 1.14E-10 | 0.278 |
| GAPIT | WSPCBLUP | AX-94284428 | 8 | 8,627,848 | 5.46E-07 | 0.225 |
| GAPIT | WSPCE1 | AX-93751921 | 8 | 8,631,913 | 9.95E-07 | 0.095 |
| GAPIT | WSPCE2 | AX-93751921 | 8 | 8,631,913 | 7.41E-07 | 0.099 |
| GAPIT | WSPCE5 | AX-93751921 | 8 | 8,631,913 | 1.76E-10 | 0.230 |
| GAPIT | WSPCE6 | AX-93751921 | 8 | 8,631,913 | 5.29E-10 | 0.225 |
| GAPIT | WSPCBLUP | AX-93751921 | 8 | 8,631,913 | 3.03E-07 | 0.257 |
| GAPIT | WSPCE5 | AX-93751923 | 8 | 8,634,985 | 4.50E-07 | 0.155 |
| GAPIT | WSPCE6 | AX-93751923 | 8 | 8,634,985 | 9.84E-09 | 0.208 |
| GAPIT | WSPCBLUP | AX-93751923 | 8 | 8,634,985 | 1.81E-06 | 0.233 |
| GAPIT | WSPCE1 | AX-94048210 | 8 | 8,643,359 | 2.22E-09 | 0.117 |
| GAPIT | WSPCE2 | AX-94048210 | 8 | 8,643,359 | 1.54E-11 | 0.121 |
| GAPIT | WSPCE3 | AX-94048210 | 8 | 8,643,359 | 1.76E-08 | 0.106 |
| GAPIT | WSPCE5 | AX-94048210 | 8 | 8,643,359 | 4.63E-14 | 0.265 |
| GAPIT | WSPCE6 | AX-94048210 | 8 | 8,643,359 | 1.75E-13 | 0.260 |
| GAPIT | WSPCBLUP | AX-94048210 | 8 | 8,643,359 | 1.57E-12 | 0.248 |
| GAPIT | WSPCE1 | AX-94284427 | 8 | 8,645,105 | 2.22E-07 | 0.117 |
| GAPIT | WSPCE2 | AX-94284427 | 8 | 8,645,105 | 1.54E-07 | 0.121 |
| GAPIT | WSPCE3 | AX-94284427 | 8 | 8,645,105 | 1.76E-06 | 0.106 |
| GAPIT | WSPCE5 | AX-94284427 | 8 | 8,645,105 | 4.63E-11 | 0.265 |
| GAPIT | WSPCE6 | AX-94284427 | 8 | 8,645,105 | 1.75E-10 | 0.260 |
| GAPIT | WSPCBLUP | AX-94284427 | 8 | 8,645,105 | 1.57E-08 | 0.248 |
| GAPIT | WSPCE1 | AX-94048212 | 8 | 8,648,879 | 7.86E-07 | 0.107 |
| GAPIT | WSPCE2 | AX-94048212 | 8 | 8,648,879 | 6.13E-07 | 0.110 |
| GAPIT | WSPCE5 | AX-94048212 | 8 | 8,648,879 | 3.27E-10 | 0.248 |
| GAPIT | WSPCE6 | AX-94048212 | 8 | 8,648,879 | 5.71E-10 | 0.248 |
| GAPIT | WSPCBLUP | AX-94048212 | 8 | 8,648,879 | 4.83E-08 | 0.245 |
| GAPIT | WSPCE1 | AX-94048214 | 8 | 8,651,335 | 3.73E-06 | 0.103 |
| GAPIT | WSPCE2 | AX-94048214 | 8 | 8,651,335 | 2.79E-06 | 0.107 |
| GAPIT | WSPCE5 | AX-94048214 | 8 | 8,651,335 | 7.97E-10 | 0.266 |
| GAPIT | WSPCE6 | AX-94048214 | 8 | 8,651,335 | 5.79E-10 | 0.270 |
| GAPIT | WSPCBLUP | AX-94048214 | 8 | 8,651,335 | 2.52E-06 | 0.245 |
| GAPIT | WSPCE1 | AX-94048288 | 8 | 8,862,259 | 6.28E-11 | 0.158 |
| GAPIT | WSPCE2 | AX-94048288 | 8 | 8,862,259 | 6.81E-11 | 0.159 |
| GAPIT | WSPCE3 | AX-94048288 | 8 | 8,862,259 | 1.12E-10 | 0.159 |
| GAPIT | WSPCE4 | AX-94048288 | 8 | 8,862,259 | 5.04E-08 | 0.117 |
| GAPIT | WSPCBLUP | AX-94048288 | 8 | 8,862,259 | 7.45E-08 | 0.091 |
| GAPIT | WSPCE1 | AX-94048289 | 8 | 8,862,952 | 6.87E-11 | 0.157 |
| GAPIT | WSPCE2 | AX-94048289 | 8 | 8,862,952 | 7.89E-11 | 0.159 |
| GAPIT | WSPCE3 | AX-94048289 | 8 | 8,862,952 | 1.34E-10 | 0.159 |
| GAPIT | WSPCE4 | AX-94048289 | 8 | 8,862,952 | 5.76E-08 | 0.117 |
| GAPIT | WSPCBLUP | AX-94048289 | 8 | 8,862,952 | 1.06E-07 | 0.091 |
| GAPIT | WSPCE1 | AX-94048290 | 8 | 8,863,028 | 6.28E-11 | 0.157 |
| GAPIT | WSPCE2 | AX-94048290 | 8 | 8,863,028 | 7.04E-11 | 0.159 |
| GAPIT | WSPCE3 | AX-94048290 | 8 | 8,863,028 | 1.22E-10 | 0.159 |
| GAPIT | WSPCE4 | AX-94048290 | 8 | 8,863,028 | 5.27E-08 | 0.117 |
| GAPIT | WSPCBLUP | AX-94048290 | 8 | 8,863,028 | 1.22E-07 | 0.088 |
| GAPIT | WSPCE1 | AX-94048322 | 8 | 8,941,023 | 1.70E-07 | 0.119 |
| GAPIT | WSPCE2 | AX-94048322 | 8 | 8,941,023 | 2.31E-07 | 0.119 |
| GAPIT | WSPCE3 | AX-94048322 | 8 | 8,941,023 | 2.52E-07 | 0.121 |
| GAPIT | WSPCE4 | AX-94048322 | 8 | 8,941,023 | 3.00E-02 | 0.102 |
| GAPIT | WSPCE1 | AX-93933852 | 10 | 40,304,375 | 4.57E-06 | 0.098 |
| GAPIT | WSPCE2 | AX-93933852 | 10 | 40,304,375 | 4.53E-06 | 0.093 |
| GAPIT | WSPCE4 | AX-93933852 | 10 | 40,304,375 | 4.02E-06 | 0.099 |
| GAPIT | WSPCE5 | AX-93933852 | 10 | 40,304,375 | 1.50E-13 | 0.240 |
| GAPIT | WSPCE6 | AX-93933852 | 10 | 40,304,375 | 2.42E-06 | 0.120 |
| GAPIT | WSPCE1 | AX-93795121 | 11 | 37,300,134 | 3.28E-07 | 0.114 |
| GAPIT | WSPCE2 | AX-93795121 | 11 | 37,300,134 | 6.31E-07 | 0.111 |
| GAPIT | WSPCE3 | AX-93795121 | 11 | 37,300,134 | 6.70E-07 | 0.113 |
| GAPIT | WSPCE4 | AX-93795121 | 11 | 37,300,134 | 8.47E-07 | 0.111 |
| GAPIT | WSPCE5 | AX-93795121 | 11 | 37,300,134 | 1.20E-06 | 0.044 |
| GAPIT | WSPCE6 | AX-93795121 | 11 | 37,300,134 | 2.48E-06 | 0.039 |
| GAPIT | WSPCE1 | AX-93805526 | 12 | 36,952,663 | 1.22E-07 | 0.110 |
| GAPIT | WSPCE2 | AX-93805526 | 12 | 36,952,663 | 4.22E-07 | 0.096 |
| GAPIT | WSPCBLUP | AX-93805526 | 12 | 36,952,663 | 2.20E-09 | 0.163 |
| GAPIT | WSPCE1 | AX-93815429 | 13 | 30,954,269 | 1.29E-07 | 0.107 |
| GAPIT | WSPCE2 | AX-93815429 | 13 | 30,954,269 | 1.38E-07 | 0.108 |
| GAPIT | WSPCE3 | AX-93815429 | 13 | 30,954,269 | 2.78E-06 | 0.088 |
| GAPIT | WSPCE4 | AX-93815429 | 13 | 30,954,269 | 1.30E-06 | 0.094 |
| GAPIT | WSPCE1 | AX-93643834 | 13 | 30,985,921 | 5.82E-08 | 0.126 |
| GAPIT | WSPCE2 | AX-93643834 | 13 | 30,985,921 | 5.78E-08 | 0.128 |
| GAPIT | WSPCE4 | AX-93643834 | 13 | 30,985,921 | 3.02E-06 | 0.103 |
| GAPIT | WSPCE1 | AX-94112235 | 13 | 30,988,071 | 3.94E-09 | 0.130 |
| GAPIT | WSPCE2 | AX-94112235 | 13 | 30,988,071 | 4.51E-09 | 0.131 |
| GAPIT | WSPCE3 | AX-94112235 | 13 | 30,988,071 | 6.35E-07 | 0.099 |
| GAPIT | WSPCE4 | AX-94112235 | 13 | 30,988,071 | 3.26E-07 | 0.104 |
| GAPIT | WSPCE1 | AX-94112254 | 13 | 31,040,000 | 1.45E-08 | 0.121 |
| GAPIT | WSPCE2 | AX-94112254 | 13 | 31,040,000 | 1.63E-08 | 0.122 |
| GAPIT | WSPCE3 | AX-94112254 | 13 | 31,040,000 | 2.23E-06 | 0.090 |
| GAPIT | WSPCE4 | AX-94112254 | 13 | 31,040,000 | 1.73E-06 | 0.092 |
| GAPIT | WSPCE5 | AX-94112358 | 13 | 31,275,903 | 4.52E-06 | 0.095 |
| GAPIT | WSPCE6 | AX-94112358 | 13 | 31,275,903 | 6.65E-07 | 0.123 |
| GAPIT | WSPCE1 | AX-94156641 | 17 | 7,962,112 | 2.01E-06 | 0.103 |
| GAPIT | WSPCE2 | AX-94156641 | 17 | 7,962,112 | 3.58E-06 | 0.100 |
| GAPIT | WSPCE3 | AX-94156641 | 17 | 7,962,112 | 2.24E-06 | 0.106 |
| GAPIT | WSPCBLUP | AX-94156641 | 17 | 7,962,112 | 2.67E-06 | 0.181 |
| GAPIT | WSPCE1 | AX-94167541 | 18 | 6,522,585 | 4.24E-06 | 0.083 |
| GAPIT | WSPCE2 | AX-94167541 | 18 | 6,522,585 | 4.37E-06 | 0.084 |
| GAPIT | WSPCE3 | AX-94167541 | 18 | 6,522,585 | 4.91E-06 | 0.062 |
| GAPIT | WSPCE4 | AX-94167541 | 18 | 6,522,585 | 3.46E-06 | 0.088 |
| GAPIT | WSPCE5 | AX-94167600 | 18 | 6,649,496 | 3.15E-06 | 0.078 |
| GAPIT | WSPCE6 | AX-93870261 | 18 | 6,745,767 | 2.59E-08 | 0.086 |
| GAPIT | WSPCE1 | AX-94170844 | 18 | 17,076,547 | 4.00E-06 | 0.082 |
| GAPIT | WSPCE2 | AX-94170844 | 18 | 17,076,547 | 4.78E-06 | 0.078 |
| GAPIT | WSPCE4 | AX-94170844 | 18 | 17,076,547 | 4.56E-06 | 0.081 |
| GAPIT | WSPCE5 | AX-93873511 | 18 | 17,332,632 | 2.41E-06 | 0.089 |
| GAPIT | WSPCE1 | AX-94196006 | 19 | 47,615,818 | 3.01E-06 | 0.101 |
| GAPIT | WSPCE2 | AX-94196006 | 19 | 47,615,818 | 1.64E-06 | 0.107 |
| GAPIT | WSPCBLUP | AX-94196006 | 19 | 47,615,818 | 3.16E-06 | 0.096 |
| GAPIT | WSPCE5 | AX-93898169 | 19 | 47,653,598 | 3.42E-09 | 0.012 |
| mrMLM | WSPCE2 | AX-93920873 | 5 | 1,002,400 | 4.610 | 0.039 |
| mrMLM | WSPCBLUP | AX-93920873 | 5 | 1,002,400 | 5.229 | 0.034 |
| mrMLM | WSPCE1 | AX-94011018 | 5 | 1,870,377 | 6.959 | 0.029 |
| mrMLM | WSPCE2 | AX-94011018 | 5 | 1,870,377 | 6.653 | 0.055 |
| mrMLM | WSPCE1 | AX-94283226 | 7 | 3,484,506 | 12.191 | 0.065 |
| mrMLM | WSPCE2 | AX-94283226 | 7 | 3,484,506 | 9.105 | 0.052 |
| mrMLM | WSPCE3 | AX-94283226 | 7 | 3,484,506 | 8.084 | 0.044 |
| mrMLM | WSPCBLUP | AX-94283226 | 7 | 3,484,506 | 6.864 | 0.035 |
| mrMLM | WSPCE5 | AX-93751650 | 8 | 7,814,032 | 15.710 | 0.158 |
| mrMLM | WSPCE6 | AX-93751650 | 8 | 7,814,032 | 4.929 | 0.065 |
| mrMLM | WSPCE1 | AX-93755074 | 8 | 17,798,726 | 4.650 | 0.016 |
| mrMLM | WSPCE4 | AX-93755074 | 8 | 17,798,726 | 8.279 | 0.063 |
| mrMLM | WSPCBLUP | AX-93755074 | 8 | 17,798,726 | 5.487 | 0.026 |
| mrMLM | WSPCE1 | AX-93758268 | 8 | 39,019,652 | 16.631 | 0.112 |
| mrMLM | WSPCE2 | AX-93758268 | 8 | 39,019,652 | 11.278 | 0.096 |
| mrMLM | WSPCE3 | AX-93758268 | 8 | 39,019,652 | 7.028 | 0.053 |
| mrMLM | WSPCBLUP | AX-93758268 | 8 | 39,019,652 | 8.576 | 0.058 |
| mrMLM | WSPCE1 | AX-93929929 | 8 | 40,184,481 | 5.227 | 0.024 |
| mrMLM | WSPCE2 | AX-93929929 | 8 | 40,184,481 | 8.804 | 0.055 |
| mrMLM | WSPCE3 | AX-93929929 | 8 | 40,184,481 | 10.430 | 0.051 |
| mrMLM | WSPCBLUP | AX-93929929 | 8 | 40,184,481 | 5.647 | 0.030 |
| mrMLM | WSPCE5 | AX-94048148 | 8 | 8,368,519 | 7.657 | 0.188 |
| mrMLM | WSPCE6 | AX-94048148 | 8 | 8,368,519 | 16.428 | 0.206 |
| mrMLM | WSPCE1 | AX-94048288 | 8 | 8,862,259 | 18.269 | 0.107 |
| mrMLM | WSPCE2 | AX-94048288 | 8 | 8,862,259 | 15.422 | 0.119 |
| mrMLM | WSPCE3 | AX-94048288 | 8 | 8,862,259 | 12.869 | 0.089 |
| mrMLM | WSPCE4 | AX-94048288 | 8 | 8,862,259 | 10.891 | 0.096 |
| mrMLM | WSPCBLUP | AX-94048288 | 8 | 8,862,259 | 9.420 | 0.057 |
| mrMLM | WSPCE2 | AX-93936122 | 11 | 35,186,027 | 4.503 | 0.024 |
| mrMLM | WSPCE3 | AX-93936122 | 11 | 35,186,027 | 4.615 | 0.023 |
| mrMLM | WSPCE4 | AX-93936122 | 11 | 35,186,027 | 5.606 | 0.043 |
| mrMLM | WSPCE1 | AX-94268787 | 12 | 2,583,421 | 7.997 | 0.043 |
| mrMLM | WSPCE2 | AX-94268787 | 12 | 2,583,421 | 8.902 | 0.051 |
| mrMLM | WSPCE3 | AX-94268787 | 12 | 2,583,421 | 3.000 | 0.023 |
| mrMLM | WSPCBLUP | AX-94268787 | 12 | 2,583,421 | 7.225 | 0.035 |
| mrMLM | WSPCE1 | AX-94112235 | 13 | 30,988,071 | 5.241 | 0.052 |
| mrMLM | WSPCE2 | AX-94112235 | 13 | 30,988,071 | 6.452 | 0.039 |
| mrMLM | WSPCE2 | AX-94150758 | 16 | 28,496,401 | 3.894 | 0.027 |
| mrMLM | WSPCE3 | AX-94150758 | 16 | 28,496,401 | 3.281 | 0.022 |
| mrMLM | WSPCE4 | AX-94150758 | 16 | 28,496,401 | 5.084 | 0.045 |
| mrMLM | WSPCBLUP | AX-94150758 | 16 | 28,496,401 | 4.346 | 0.024 |
| mrMLM | WSPCE1 | AX-94152761 | 16 | 33,855,036 | 5.871 | 0.042 |
| mrMLM | WSPCE3 | AX-94152761 | 16 | 33,855,036 | 4.603 | 0.033 |
| mrMLM | WSPCE4 | AX-93652054 | 17 | 13,682,702 | 5.141 | 0.043 |
| mrMLM | WSPCBLUP | AX-93652054 | 17 | 13,682,702 | 3.536 | 0.020 |
| mrMLM | WSPCE1 | AX-94156641 | 17 | 7,962,112 | 4.755 | 0.172 |
| mrMLM | WSPCE2 | AX-94156641 | 17 | 7,962,112 | 3.185 | 0.068 |
| mrMLM | WSPCE3 | AX-94156641 | 17 | 7,962,112 | 9.906 | 0.229 |
| mrMLM | WSPCBLUP | AX-94156641 | 17 | 7,962,112 | 11.144 | 0.223 |
| mrMLM | WSPCE1 | AX-93883121 | 18 | 56,877,979 | 5.513 | 0.047 |
| mrMLM | WSPCE3 | AX-93883121 | 18 | 56,877,979 | 3.027 | 0.024 |
| mrMLM | WSPCE3 | AX-93956947 | 20 | 35,095,841 | 3.329 | 0.014 |
| mrMLM | WSPCBLUP | AX-93956947 | 20 | 35,095,841 | 3.317 | 0.019 |
| mrMLM | WSPCE4 | AX-94198826 | 20 | 5,840,087 | 4.189 | 0.041 |
| mrMLM | WSPCBLUP | AX-94198826 | 20 | 5,840,087 | 7.612 | 0.049 |
| mrMLM | WSPCE1 | AX-94203096 | 20 | 28,261,212 | 8.279 | 0.039 |
| mrMLM | WSPCE2 | AX-94203096 | 20 | 28,261,212 | 7.855 | 0.049 |
| ISIS EM-BLASSO | WSPCE5 | AX-94271709 | 3 | 40,325,183 | 5.914 | 0.014 |
| ISIS EM-BLASSO | WSPCE6 | AX-94271709 | 3 | 40,325,183 | 3.443 | 0.009 |
| ISIS EM-BLASSO | WSPCE1 | AX-93703986 | 4 | 4,451,706 | 5.356 | 0.018 |
| ISIS EM-BLASSO | WSPCE2 | AX-93703986 | 4 | 4,451,706 | 6.604 | 0.023 |
| ISIS EM-BLASSO | WSPCE2 | AX-94283226 | 7 | 3,484,506 | 6.384 | 0.020 |
| ISIS EM-BLASSO | WSPCBLUP | AX-94283226 | 7 | 3,484,506 | 4.364 | 0.009 |
| ISIS EM-BLASSO | WSPCE1 | AX-93749510 | 8 | 1,964,081 | 4.793 | 0.013 |
| ISIS EM-BLASSO | WSPCE2 | AX-93749510 | 8 | 1,964,081 | 6.237 | 0.022 |
| ISIS EM-BLASSO | WSPCE3 | AX-93749510 | 8 | 1,964,081 | 4.530 | 0.024 |
| ISIS EM-BLASSO | WSPCE4 | AX-93749510 | 8 | 1,964,081 | 3.195 | 0.012 |
| ISIS EM-BLASSO | WSPCE1 | AX-93755074 | 8 | 17,798,726 | 8.633 | 0.023 |
| ISIS EM-BLASSO | WSPCE3 | AX-93755074 | 8 | 17,798,726 | 7.062 | 0.042 |
| ISIS EM-BLASSO | WSPCE4 | AX-93755074 | 8 | 17,798,726 | 4.490 | 0.019 |
| ISIS EM-BLASSO | WSPCBLUP | AX-93755074 | 8 | 17,798,726 | 11.048 | 0.028 |
| ISIS EM-BLASSO | WSPCE1 | AX-93758268 | 8 | 39,019,652 | 9.363 | 0.042 |
| ISIS EM-BLASSO | WSPCE2 | AX-93758268 | 8 | 39,019,652 | 6.511 | 0.032 |
| ISIS EM-BLASSO | WSPCE3 | AX-93758268 | 8 | 39,019,652 | 5.417 | 0.043 |
| ISIS EM-BLASSO | WSPCE1 | AX-93758680 | 8 | 40,192,377 | 10.966 | 0.036 |
| ISIS EM-BLASSO | WSPCE3 | AX-93758680 | 8 | 40,192,377 | 9.495 | 0.065 |
| ISIS EM-BLASSO | WSPCE2 | AX-93929929 | 8 | 40,184,481 | 9.791 | 0.036 |
| ISIS EM-BLASSO | WSPCBLUP | AX-93929929 | 8 | 40,184,481 | 6.000 | 0.015 |
| ISIS EM-BLASSO | WSPCE5 | AX-94048086 | 8 | 8,204,675 | 3.207 | 0.015 |
| ISIS EM-BLASSO | WSPCBLUP | AX-94048086 | 8 | 8,204,675 | 16.573 | 0.087 |
| ISIS EM-BLASSO | WSPCE1 | AX-94048288 | 8 | 8,862,259 | 15.834 | 0.069 |
| ISIS EM-BLASSO | WSPCE2 | AX-94048288 | 8 | 8,862,259 | 15.226 | 0.072 |
| ISIS EM-BLASSO | WSPCE3 | AX-94048288 | 8 | 8,862,259 | 10.863 | 0.100 |
| ISIS EM-BLASSO | WSPCE4 | AX-94048288 | 8 | 8,862,259 | 8.918 | 0.050 |
| ISIS EM-BLASSO | WSPCBLUP | AX-94048288 | 8 | 8,862,259 | 13.416 | 0.049 |
| ISIS EM-BLASSO | WSPCE1 | AX-93936122 | 11 | 35,186,027 | 2.675 | 0.005 |
| ISIS EM-BLASSO | WSPCE2 | AX-93936122 | 11 | 35,186,027 | 3.078 | 0.006 |
| ISIS EM-BLASSO | WSPCE4 | AX-93936122 | 11 | 35,186,027 | 4.092 | 0.014 |
| ISIS EM-BLASSO | WSPCBLUP | AX-93936122 | 11 | 35,186,027 | 2.876 | 0.006 |
| ISIS EM-BLASSO | WSPCE4 | AX-93804981 | 12 | 35,602,425 | 4.828 | 0.019 |
| ISIS EM-BLASSO | WSPCBLUP | AX-93804981 | 12 | 35,602,425 | 7.881 | 0.020 |
| ISIS EM-BLASSO | WSPCE1 | AX-93649393 | 15 | 48,823,741 | 9.620 | 0.065 |
| ISIS EM-BLASSO | WSPCE2 | AX-93649393 | 15 | 48,823,741 | 7.496 | 0.056 |
| ISIS EM-BLASSO | WSPCE3 | AX-93649393 | 15 | 48,823,741 | 4.065 | 0.053 |
| ISIS EM-BLASSO | WSPCE1 | AX-94156641 | 17 | 7,962,112 | 6.579 | 0.078 |
| ISIS EM-BLASSO | WSPCE2 | AX-94156641 | 17 | 7,962,112 | 4.507 | 0.059 |
| ISIS EM-BLASSO | WSPCE1 | AX-94180740 | 18 | 56,891,288 | 5.631 | 0.016 |
| ISIS EM-BLASSO | WSPCE2 | AX-94180740 | 18 | 56,891,288 | 3.592 | 0.010 |
| ISIS EM-BLASSO | WSPCE1 | AX-94182257 | 18 | 60,470,813 | 2.648 | 0.005 |
| ISIS EM-BLASSO | WSPCE3 | AX-94182257 | 18 | 60,470,813 | 3.815 | 0.018 |
| ISIS EM-BLASSO | WSPCBLUP | AX-94182257 | 18 | 60,470,813 | 5.031 | 0.011 |
| ISIS EM-BLASSO | WSPCE2 | AX-93897507 | 19 | 45,738,834 | 5.397 | 0.013 |
| ISIS EM-BLASSO | WSPCBLUP | AX-93897507 | 19 | 45,738,834 | 3.145 | 0.003 |
| ISIS EM-BLASSO | WSPCE2 | AX-94198333 | 20 | 4,049,700 | 5.643 | 0.018 |
| ISIS EM-BLASSO | WSPCE3 | AX-94198333 | 20 | 4,049,700 | 4.994 | 0.032 |
| ISIS EM-BLASSO | WSPCE4 | AX-94198333 | 20 | 4,049,700 | 4.010 | 0.016 |

Table S4 Epistatic loci for soybean protein content (PC) and water soluble protein content (WSPC) across six environments in natural population by EGWAS.

| **Traits** | **SNP1** | **Chr.** | **Position** | **SNP2** | **Chr.** | **Position** | ***P* value** |
| --- | --- | --- | --- | --- | --- | --- | --- |
| PC1 | AX-93995056 | 3 | 37773722 | AX-93658530 | 19 | 38139413 | 1.141E-24 |
| PC3 | AX-93995056 | 3 | 37773722 | AX-94073682 | 10 | 10894995 | 1.314E-06 |
| PC4 | AX-93995056 | 3 | 37773722 | AX-94289127 | 16 | 3000315 | 1.107E-06 |
| PcBLUP | AX-93995056 | 3 | 37773722 | AX-93658530 | 19 | 38139413 | 2.896E-17 |
| PC1 | AX-93698777 | 3 | 37681962 | AX-93658530 | 19 | 38139413 | 9.05E-27 |
| PC3 | AX-93698777 | 3 | 37681962 | AX-94073682 | 10 | 10894995 | 5.721E-07 |
| PC4 | AX-93698777 | 3 | 37681962 | AX-93658530 | 19 | 38139413 | 7.613E-07 |
| PcBLUP | AX-93698777 | 3 | 37681962 | AX-93658530 | 19 | 38139413 | 2.284E-18 |
| PC3 | AX-93736753 | 6 | 48107147 | AX-93900712 | 20 | 4642063 | 3.371E-07 |
| PC3 | AX-93748464 | 7 | 43807170 | AX-93736820 | 6 | 48280223 | 1.602E-06 |
| PC4 | AX-93748465 | 7 | 43807170 | AX-94144257 | 6 | 48280223 | 7.602E-07 |
| PC1 | AX-93822697 | 14 | 7715347 | AX-93952504 | 18 | 56107761 | 1.771E-29 |
| PC5 | AX-93822697 | 14 | 7715347 | AX-93952504 | 18 | 56107761 | 1.33E-14 |
| PcBLUP | AX-93822697 | 14 | 7715347 | AX-93952504 | 18 | 56107761 | 2.33E-16 |
| WSPC1 | AX-93995781 | 3 | 39582595 | AX-93809258 | 13 | 7046205 | 1.23E-16 |
| WSPC3 | AX-93995781 | 3 | 39582595 | AX-93809258 | 13 | 7046205 | 9.104E-07 |
| WSPC4 | AX-93996119 | 3 | 40433979 | AX-93809258 | 13 | 7046205 | 4.594E-06 |
| WSPC1 | AX-94048155 | 8 | 8386661 | AX-93937867 | 12 | 34980811 | 1.533E-06 |
| WSPC2 | AX-94048155 | 8 | 8386661 | AX-93937867 | 12 | 34980811 | 1.971E-06 |
| WSPCBLUP | AX-94048155 | 8 | 8386661 | AX-94091153 | 11 | 35518790 | 3.687E-06 |
| WSPC1 | AX-93751882 | 8 | 8422602 | AX-93882799 | 18 | 56122678 | 1.576E-06 |
| WSPC2 | AX-93751882 | 8 | 8422602 | AX-93740126 | 7 | 6596974 | 2.864E-06 |
| WSPC6 | AX-93751882 | 8 | 8422602 | AX-94019308 | 5 | 38453905 | 9.884E-07 |
| WSPC1 | AX-94048176 | 8 | 8446615 | AX-93823045 | 14 | 8605167 | 3.043E-06 |
| WSPC2 | AX-94048176 | 8 | 8446615 | AX-93823045 | 14 | 8605167 | 3.885E-06 |
| WSPC4 | AX-94048176 | 8 | 8446615 | AX-94037820 | 7 | 10182704 | 5.052E-07 |
| WSPCBLUP | AX-94048176 | 8 | 8446615 | AX-93882799 | 18 | 56122678 | 4.536E-07 |
| WSPC1 | AX-93751901 | 8 | 8500850 | AX-93652182 | 17 | 15899194 | 2.035E-07 |
| WSPC2 | AX-93751901 | 8 | 8500850 | AX-93652182 | 17 | 15899194 | 2.684E-07 |
| WSPCBLUP | AX-93751901 | 8 | 8500850 | AX-93652182 | 17 | 15899194 | 9.952E-07 |
| WSPC1 | AX-93751903 | 8 | 8532575 | AX-93652182 | 17 | 15899194 | 2.035E-07 |
| WSPC2 | AX-93751903 | 8 | 8532575 | AX-93652182 | 17 | 15899194 | 2.684E-07 |
| WSPCBLUP | AX-93751903 | 8 | 8532575 | AX-93652182 | 17 | 15899194 | 9.952E-07 |
| WSPC1 | AX-94048210 | 8 | 8643359 | AX-93652182 | 17 | 15899194 | 2.035E-07 |
| WSPC2 | AX-94048210 | 8 | 8643359 | AX-93652182 | 17 | 15899194 | 2.684E-07 |
| WSPCBLUP | AX-94048210 | 8 | 8643359 | AX-93652182 | 17 | 15899194 | 9.952E-07 |
| WSPC1 | AX-93755074 | 8 | 17798726 | AX-93617393 | 1 | 50711950 | 5.031E-07 |
| WSPC2 | AX-93755074 | 8 | 17798726 | AX-93617393 | 1 | 50711950 | 3.565E-07 |
| WSPCBLUP | AX-93755074 | 8 | 17798726 | AX-93617393 | 1 | 50711950 | 5.472E-07 |
| WSPC3 | AX-93755074 | 8 | 17798726 | AX-93987734 | 1 | 50711950 | 4.552E-06 |
| WSPC1 | AX-94051376 | 8 | 17805925 | AX-93617393 | 1 | 50711950 | 2.838E-07 |
| WSPC3 | AX-94051376 | 8 | 17805925 | AX-93617393 | 1 | 50711950 | 2.793E-06 |
| WSPC1 | AX-94079691 | 10 | 43174460 | AX-93769439 | 9 | 35063154 | 7.215E-07 |
| WSPC2 | AX-94079691 | 10 | 43174460 | AX-93769439 | 9 | 35063154 | 1.663E-06 |
| WSPC3 | AX-94079691 | 10 | 43174460 | AX-93769439 | 9 | 35063154 | 2.929E-06 |
| WSPC3 | AX-93939961 | 13 | 30951300 | AX-93617903 | 1 | 790446 | 2.173E-06 |
| WSPC4 | AX-93939961 | 13 | 30951300 | AX-93880615 | 18 | 50795227 | 1.379E-06 |
| WSPC2 | AX-93939961 | 13 | 30951300 | AX-93961753 | 1 | 2160318 | 2.057E-06 |
| WSPC2 | AX-94196006 | 19 | 47615818 | AX-93753563 | 8 | 13456181 | 2.105E-06 |
| WSPC3 | AX-94196006 | 19 | 47615818 | AX-93753563 | 8 | 13456181 | 2.586E-07 |
| WSPC4 | AX-94196006 | 19 | 47615818 | AX-93753563 | 8 | 13456181 | 9.226E-07 |

Table S5 QTL list for soybean protein content (PC) and water soluble protein content (WSPC) across four environments and the BLUP by QTL linkage mapping in RIL population

| **Methods** | **TraitName** | **Chromosome** | **Position** | **LeftMarker** | **RightMarker** | **LOD** | **PVE(%)** | **Add** |
| --- | --- | --- | --- | --- | --- | --- | --- | --- |
| ICIM | *q3PC2012* | 3 | 82 | Marker950668 | Marker977935 | 5.54 | 13.3371 | -0.4836 |
| ICIM | *q8PC2012* | 8 | 0 | Marker2684141 | Marker2728565 | 2.73 | 5.976 | 0.3252 |
| ICIM | *q11PC2012* | 11 | 112 | Marker812042 | Marker837694 | 2.76 | 6.1279 | -0.3258 |
| ICIM | *q19PC2012* | 19 | 2 | Marker1050238 | Marker1066605 | 3.30 | 7.3276 | -0.3557 |
| ICIM | *q3PC2013* | 3 | 82 | Marker950668 | Marker977935 | 3.82 | 8.1799 | -0.417 |
| ICIM | *q5PC2013* | 5 | 46 | Marker1806161 | Marker1910893 | 3.47 | 7.3855 | 0.3948 |
| ICIM | *q10PC2013* | 10 | 15 | Marker638369 | Marker658316 | 3.09 | 6.6149 | 0.63823 |
| ICIM | *q11PC2013* | 11 | 116 | Marker799502 | Marker844658 | 2.81 | 6.5536 | -0.3708 |
| ICIM | *q13PC2013* | 13 | 25 | Marker1793867 | Marker1714063 | 3.45 | 7.0717 | 0.3851 |
| ICIM | *q3PC2014* | 3 | 82 | Marker950668 | Marker977935 | 5.80 | 14.347 | -0.5119 |
| ICIM | *q5PC2014* | 5 | 45 | Marker1912544 | Marker1913462 | 2.70 | 6.1081 | 0.3332 |
| ICIM | *q11PC2014* | 11 | 127 | Marker785404 | Marker803779 | 3.32 | 7.5463 | -0.3685 |
| ICIM | *q19PC2014* | 19 | 3 | Marker1050238 | Marker1066605 | 3.35 | 7.7746 | -0.3754 |
| ICIM | *q3PC2015* | 3 | 82 | Marker950668 | Marker977935 | 2.57 | 5.3364 | -0.3431 |
| ICIM | *q10PC2015* | 10 | 15 | Marker638369 | Marker658316 | 5.23 | 11.4184 | 0.5115 |
| ICIM | *q12PC2015* | 12 | 12 | Marker193187 | Marker259861 | 3.96 | 8.27 | 0.4275 |
| ICIM | *q13PC2015* | 13 | 25 | Marker1793867 | Marker1714063 | 2.76 | 5.6116 | 0.3492 |
| ICIM | *q19PC2015* | 19 | 2 | Marker1050238 | Marker1066605 | 2.83 | 5.799 | -0.3559 |
| ICIM | *q3BLUPPC* | 3 | 82 | Marker950668 | Marker977935 | 7.28 | 17.136 | -0.5378 |
| ICIM | *q5BLUPPC* | 5 | 45 | Marker1912544 | Marker1913462 | 3.32 | 7.1272 | 0.3464 |
| ICIM | *q10BLUPPC* | 10 | 15 | Marker638369 | Marker658316 | 4.11 | 9.28 | 0.68823 |
| ICIM | *q11BLUPPC* | 11 | 126 | Marker802619 | Marker785404 | 3.99 | 8.5179 | -0.3769 |
| ICIM | *q19BLUPPC* | 19 | 3 | Marker1050238 | Marker1066605 | 2.86 | 6.2252 | -0.3235 |
| ICIM | *q8WSPC2012* | 8 | 106 | Marker2696788 | Marker2699628 | 2.99 | 8.2919 | 0.5332 |
| ICIM | *q12WSPC2012* | 12 | 10 | Marker249870 | Marker181786 | 3.22 | 8.8109 | 0.5513 |
| ICIM | *q3WSPC2013* | 3 | 8 | Marker878118 | Marker941301 | 3.27 | 7.5228 | 0.7419 |
| ICIM | *q8WSPC2013* | 8 | 108 | Marker2607468 | Marker2603004 | 4.28 | 15.6093 | 1.18423 |
| ICIM | *q9WSPC2013* | 9 | 53 | Marker1564423 | Marker1543731 | 3.44 | 7.6461 | 0.7493 |
| ICIM | *q8WSPC2014* | 8 | 107 | Marker2696788 | Marker2699628 | 7.96 | 21.3604 | 1.3112 |
| ICIM | *q10WSPC2014* | 10 | 30 | Marker708533 | Marker628143 | 2.62 | 6.7627 | 0.47414 |
| ICIM | *q3WSPC2015* | 3 | 8 | Marker878118 | Marker941301 | 2.50 | 5.5332 | 0.6577 |
| ICIM | *q8WSPC2015* | 8 | 107 | Marker2696788 | Marker2699628 | 8.66 | 21.2674 | 1.2819 |
| ICIM | *q10WSPC2015* | 10 | 30 | Marker708533 | Marker628143 | 2.98 | 6.991 | 0.47399 |
| ICIM | *q8BLUPWSPC* | 8 | 107 | Marker2696788 | Marker2699628 | 9.04 | 23.1413 | 0.8727 |
| ICIM | *q10BLUPWSPC* | 10 | 30 | Marker708533 | Marker628143 | 2.87 | 7.0631 | 0.4872 |
| GCIM | *q9PC2012* | 9 | 20.256 | Marker1614684 | Marker1595567 | 2.75 | 7.5593 | 0.3687 |
| GCIM | *q11PC2012* | 11 | 77.1867 | Marker804554 | Marker861597 | 6.27 | 19.1333 | -0.5866 |
| GCIM | *q3PC2013* | 3 | 16.7175 | Marker872461 | Marker922012 | 4.11 | 11.2608 | -0.4545 |
| GCIM | *q11PC2013* | 11 | 76.467 | Marker816161 | Marker861597 | 3.70 | 9.7458 | -0.4228 |
| GCIM | *q17PC2013* | 17 | 83.918 | Marker2024890 | Marker2029746 | 3.05 | 7.1618 | 0.3624 |
| GCIM | *q9PC2014* | 9 | 20.256 | Marker1614684 | Marker1595567 | 2.60 | 7.1085 | 0.3578 |
| GCIM | *q11PC2014* | 11 | 77.1867 | Marker804554 | Marker861597 | 6.45 | 19.7187 | -0.596 |
| GCIM | *q8PC2015* | 8 | 0 | Marker2677687 | Marker2684141 | 2.75 | 5.6602 | 0.3155 |
| GCIM | *q11PC2015* | 11 | 77.1867 | Marker804554 | Marker861597 | 4.04 | 17.7015 | -0.558 |
| GCIM | *q9BLUPPC* | 9 | 20.256 | Marker1614684 | Marker1595567 | 2.63 | 7.0661 | 0.3423 |
| GCIM | *q11BLUPPC* | 11 | 77.1867 | Marker804554 | Marker861597 | 6.95 | 21.0607 | -0.591 |
| GCIM | *q8WSPC2012* | 8 | 109.206 | Marker2607468 | Marker2668565 | 3.94 | 13.3496 | 0.6462 |
| GCIM | *q1WSPC2013* | 1 | 1.844 | Marker430744 | Marker433677 | 3.93 | 9.1301 | 0.8496 |
| GCIM | *q3WSPC2013* | 3 | 178.31 | Marker869715 | Marker911716 | 3.07 | 7.3409 | 0.7618 |
| GCIM | *q8WSPC2013* | 8 | 109.206 | Marker2607468 | Marker2668565 | 6.60 | 16.1928 | 1.1314 |
| GCIM | *q18WSPC2013* | 18 | 129.537 | Marker2279520 | Marker2234182 | 3.35 | 7.9498 | 0.7928 |
| GCIM | *q8WSPC2014* | 8 | 107.787 | Marker2723780 | Marker2699628 | 6.63 | 21.3736 | 1.2955 |
| GCIM | *q3WSPC2015* | 3 | 8.908 | Marker925905 | Marker934064 | 3.32 | 8.1042 | -0.8171 |
| GCIM | *q3WSPC2015* | 3 | 137.079 | Marker948137 | Marker974279 | 2.83 | 6.7844 | 0.7476 |
| GCIM | *q8WSPC2015* | 8 | 108.4965 | Marker2607468 | Marker2699628 | 8.52 | 23.2633 | 1.3843 |
| GCIM | *q1BLUPWSPC* | 1 | 1.844 | Marker430744 | Marker433677 | 4.15 | 9.9243 | 0.594 |
| GCIM | *q3BLUPWSPC* | 3 | 137.6635 | Marker977935 | Marker974279 | 2.89 | 6.6676 | 0.4869 |
| GCIM | *q8BLUPWSPC* | 8 | 108.4965 | Marker2607468 | Marker2699628 | 9.98 | 25.9758 | 0.9611 |

Table S6 Epistatic loci for soybean protein content (PC) and water soluble protein content (WSPC) across four environments in RIL population.

| **Trait Name** | **Chr.1** | **Position1** | **LeftMarker1** | **RightMarker1** | **Chr.2** | **Position2** | **LeftMarker2** | **RightMarker2** | **LOD** | **PVE(%)** | **Add1** | **Add2** | **AddbyAdd** |
| --- | --- | --- | --- | --- | --- | --- | --- | --- | --- | --- | --- | --- | --- |
| BLUPWSPC | 1 | 85 | Marker360390 | Marker312928 | 16 | 80 | Marker1157771 | Marker1142191 | 5.46 | 13.34 | 0.13 | 0.09 | 0.66 |
| BLUPWSPC | 1 | 15 | Marker399740 | Marker405817 | 8 | 105 | Marker2720519 | Marker2653911 | 5.17 | 15.39 | 0.28 | 0.10 | 0.63 |
| WSPC2012 | 1 | 40 | Marker381433 | Marker445343 | 1 | 45 | Marker381433 | Marker445343 | 4.44 | 26.01 | -1.25 | 1.18 | -1.19 |
| PC2012 | 2 | 15 | Marker2346532 | Marker2430027 | 20 | 45 | Marker1496332 | Marker1436835 | 4.76 | 10.10 | 0.09 | 0.08 | 0.42 |
| WSPC2012 | 2 | 30 | Marker2448376 | Marker2398952 | 2 | 35 | Marker2414238 | Marker2383634 | 5.50 | 27.67 | 1.06 | -1.02 | -2.01 |
| PC2014 | 4 | 25 | Marker131379 | Marker17221 | 12 | 40 | Marker227770 | Marker181842 | 4.36 | 7.83 | 0.09 | 0.01 | 0.37 |
| WSPC2012 | 4 | 5 | Marker41910 | Marker131379 | 4 | 10 | Marker131379 | Marker17221 | 4.04 | 21.24 | 1.60 | -1.68 | -1.69 |
| WSPC2012 | 4 | 5 | Marker41910 | Marker131379 | 7 | 50 | Marker2532395 | Marker2551075 | 4.01 | 21.44 | -1.59 | 1.62 | -1.90 |
| WSPC2013 | 4 | 80 | Marker53485 | Marker57689 | 7 | 115 | Marker2580623 | Marker2500377 | 4.12 | 8.91 | -0.10 | 0.17 | 0.81 |
| WSPC2015 | 4 | 5 | Marker41910 | Marker131379 | 14 | 60 | Marker562393 | Marker489485 | 4.53 | 11.18 | 0.04 | -0.16 | -0.89 |
| PC2013 | 5 | 5 | Marker1922814 | Marker1839886 | 17 | 70 | Marker2013875 | Marker1976895 | 4.49 | 10.13 | -0.10 | 0.14 | -0.44 |
| WSPC2012 | 6 | 115 | Marker2091698 | Marker2126298 | 6 | 120 | Marker2123100 | Marker2162182 | 5.11 | 22.43 | 1.38 | -1.42 | -1.54 |
| PC2014 | 7 | 5 | Marker2524720 | Marker2524164 | 18 | 105 | Marker2224263 | Marker2228155 | 4.12 | 8.02 | 0.05 | -0.05 | 0.38 |
| WSPC2014 | 7 | 95 | Marker2525913 | Marker2522046 | 13 | 60 | Marker1766807 | Marker1736710 | 4.34 | 12.91 | 0.44 | 0.14 | 0.90 |
| WSPC2015 | 7 | 50 | Marker2532395 | Marker2551075 | 14 | 75 | Marker469358 | Marker553980 | 4.76 | 8.93 | 0.12 | -0.14 | -0.81 |
| PC2012 | 10 | 15 | Marker638369 | Marker658316 | 13 | 5 | Marker1754560 | Marker1778051 | 4.83 | 15.74 | 0.17 | 0.21 | 0.48 |
| PC2014 | 10 | 30 | Marker708533 | Marker628143 | 14 | 115 | Marker544006 | Marker452662 | 4.07 | 9.33 | 0.12 | 0.17 | 0.38 |
| WSPC2012 | 11 | 40 | Marker820747 | Marker850559 | 11 | 45 | Marker820747 | Marker850559 | 4.13 | 26.13 | -1.29 | 1.12 | -1.42 |
| PC2012 | 12 | 100 | Marker211041 | Marker164759 | 19 | 65 | Marker994902 | Marker1095688 | 4.17 | 11.33 | -0.09 | -0.05 | 0.43 |
| WSPC2012 | 12 | 25 | Marker196229 | Marker198209 | 12 | 30 | Marker196229 | Marker198209 | 5.20 | 27.67 | 1.32 | -1.30 | -2.16 |
| BLUPWSPC | 16 | 75 | Marker1152652 | Marker1181762 | 18 | 90 | Marker2250359 | Marker2291925 | 4.26 | 9.59 | 0.10 | -0.05 | 0.54 |
| PC2012 | 16 | 10 | Marker1148194 | Marker1154323 | 18 | 110 | Marker2260624 | Marker2304170 | 4.82 | 10.44 | -0.18 | -0.10 | 0.42 |
| WSPC2012 | 17 | 45 | Marker1989171 | Marker2014517 | 17 | 50 | Marker2014517 | Marker2027734 | 5.64 | 23.81 | 1.41 | -1.28 | -1.95 |

Table S7 Expression pattern of the candidate genes underlying the loci at seed developmental stages

| **Candidate genes** | **young_leaf** | **flower** | **one cm pod** | **pod shell 10DAF** | **pod shell 14DAF** | **seed 10DAF** | **seed 14DAF** | **seed 21DAF** | **seed 25DAF** | **seed 28DAF** | **seed 35DAF** | **seed 42DAF** | **root** | **nodule** |
| --- | --- | --- | --- | --- | --- | --- | --- | --- | --- | --- | --- | --- | --- | --- |
| *Glyma.03G156100* | 25 | 24 | 25 | 20 | 19 | 22 | 15 | 7 | 14 | 8 | 11 | 9 | 27 | 3 |
| *Glyma.05G070600* | 7 | 72 | 4 | 5 | 5 | 2 | 3 | 3 | 14 | 11 | 39 | 29 | 87 | 10 |
| *Glyma.06G220300* | (3.15) | (0.09) | (2.77) | | | (1.95) | | | | | | | (2.14) | (1.29) |
| *Glyma.09G087200* | (52.23) | (6.77) | (8.58) | | | (6.23) | | | | | | | (0.15) | (1.41) |
| *Glyma.10G177000* | 73 | 26 | 26 | 35 | 31 | 11 | 6 | 3 | 13 | 7 | 13 | 1 | 5 | 2 |
| *Glyma.11G234600* | 18 | 77 | 56 | 46 | 43 | 12 | 14 | 4 | 8 | 13 | 22 | 19 | 73 | 11 |
| *Glyma.12G179700* | 4 | 2 | 27 | 23 | 26 | 6 | 26 | 7 | 9 | 5 | 4 | 0 | 11 | 0 |
| *Glyma.13G123500* | 0 | 0 | 0 | 0 | 0 | 0 | 0 | 0 | 5591 | 15500 | 92982 | 130315 | 0 | 0 |
| *Glyma.15G098100* | 77 | 440 | 105 | 300 | 239 | 67 | 76 | 39 | 77 | 49 | 53 | 12 | 72 | 656 |
| *Glyma.15G138200* | 50 | 54 | 56 | 47 | 43 | 52 | 31 | 12 | 18 | 14 | 17 | 15 | 53 | 53 |
| *Glyma.19G236600* | 0 | 0 | 0 | 0 | 0 | 0 | 0 | 0 | 3 | 3 | 53 | 355 | 0 | 0 |
| *Glyma.08G112300* | 637 | 469 | 498 | 308 | 181 | 333 | 271 | 168 | 152 | 139 | 128 | 50 | 174 | 45 |
| *Glyma.08G113400* | 32 | 109 | 17 | 25 | 47 | 45 | 3 | 4 | 4 | 2 | 9 | 4 | 65 | 10 |
| *Glyma.10G177000* | 73 | 26 | 26 | 35 | 31 | 11 | 6 | 3 | 13 | 7 | 13 | 1 | 5 | 2 |
| *Glyma.11G234600* | 18 | 77 | 56 | 46 | 43 | 12 | 14 | 4 | 8 | 13 | 22 | 19 | 73 | 11 |
| *Glyma.12G205100* | (4.46) | (0.40) | (0.25) | | | (0.73) | | | | | | | (0) | (0.07) |
| *Glyma.13G194400* | 0 | 0 | 0 | 0 | 0 | 2 | 20 | 30 | 725 | 645 | 3150 | 2129 | 0 | 0 |
| *Glyma.17G097800* | 62 | 95 | 56 | 66 | 54 | 35 | 55 | 24 | 54 | 36 | 70 | 34 | 52 | 40 |
| *Glyma.18G071900* | 0 | 42 | 71 | 14 | 14 | 13 | 3 | 1 | 0 | 0 | 0 | 0 | 11 | 2 |
| *Glyma.18G127100* | 0 | 0 | 2 | 0 | 0 | 6 | 7 | 5 | 2 | 1 | 1 | 0 | 0 | 0 |
| *Glyma.18G126900* | (0) | (0) | (0.01) | | | (0.03) | | | | | | | (0) | (0) |
| *Glyma.19G236600* | 0 | 0 | 0 | 0 | 0 | 0 | 0 | 0 | 3 | 3 | 53 | 355 | 0 | 0 |

Note: The expressional datasets of these candidate genes at seed developmental stages were downloaded at the website (https://phytozome.jgi.doe.gov/pz/ portal.html#!info?alias=Org_Gmax and https://soybase.org/ soyseq/).

The digital represents the expression counts of the uniquely mappable reads based on the website (https://soybase.org/soyseq/), and the digital in the brackets represents the FPKM based on the website (https://phytozome.jgi.doe.gov/pz/portal.html#!info?alias=Org_Gmax).
